# Recovery of oxidized two-dimensional MXenes through high frequency nanoscale electromechanical vibration

**DOI:** 10.1038/s41467-022-34699-3

**Published:** 2023-01-03

**Authors:** Heba Ahmed, Hossein Alijani, Ahmed El-Ghazaly, Joseph Halim, Billy J. Murdoch, Yemima Ehrnst, Emily Massahud, Amgad R. Rezk, Johanna Rosen, Leslie Y. Yeo

**Affiliations:** 1 Micro/Nanophysics Research Laboratory, School of Engineering, RMIT University, Melbourne, VIC 3000 Australia; 2 Materials Design Division, Department of Physics, Chemistry, and Biology (IFM), Linköping University, Linköping, SE-58183 Sweden; 3 RMIT Microscopy and Microanalysis Facility, RMIT University, Melbourne, VIC 3000 Australia

**Keywords:** Two-dimensional materials, Batteries

## Abstract

MXenes hold immense potential given their superior electrical properties. The practical adoption of these promising materials is, however, severely constrained by their oxidative susceptibility, leading to significant performance deterioration and lifespan limitations. Attempts to preserve MXenes have been limited, and it has not been possible thus far to reverse the material’s performance. In this work, we show that subjecting oxidized micron or nanometer thickness dry MXene films—even those constructed from nanometer-order solution-dispersed oxidized flakes—to just one minute of 10 MHz nanoscale electromechanical vibration leads to considerable removal of its surface oxide layer, whilst preserving its structure and characteristics. Importantly, electrochemical performance is recovered close to that of their original state: the pseudocapacitance, which decreased by almost 50% due to its oxidation, reverses to approximately 98% of its original value, with good capacitance retention ( ≈ 93%) following 10,000 charge–discharge cycles at 10 A g^−1^. These promising results allude to the exciting possibility for rejuvenating the material for reuse, therefore offering a more economical and sustainable route that improves its potential for practical translation.

## Abstract (web summary)

Despite their vast potential, the practical deployment of MXenes has been hampered by their tendency to be oxidized. Here, the authors show that simply vibrating MXene films in just a minute can remove the oxide layer formed and restore their electrochemical performance close to its original state.

## Introduction

MXenes are a family of two-dimensional (2D) transition metal carbides and nitrides of composition M_n+1_X_n_T_z_ (M represents an early transition metal, X carbon and/or nitrogen, and T surface termination groups such as O, OH, F and/or Cl, with n = 1, 2, 3, and z the number of terminal groups) with vastly superior and versatile intrinsic properties^1^. Besides possessing very high electrical conductivity, outstanding electrochemical capacitance, optical transparency, tunable plasmonic characteristics, good mechanical strength, and thermal stability, MXenes are hydrophilic and therefore colloidally stable in aqueous solutions, thus allowing for sustainable processing^2–6^. Given their elasticity^7^, MXenes can also be fabricated into flexible films^8–10^. As such, MXenes have shown tremendous potential for applications as diverse as energy storage^11,12^, electromagnetic interference shielding^13,14^, energy conversion^15^, catalysis^16^, wireless transmission^17,18^, sensors^19,20^, membrane separation and environmental remediation^21,22^, soft actuators^23^, and optoelectronics^24^, among others.

Despite their promise, MXenes are prone to severe oxidative degradation relatively quickly, having been reported to form oxides (e.g., titanium dioxide (TiO_2_) in the case of Ti-based MXenes) over just several hours to days when exposed to either humid air or aqueous environments^25–30^. This leads to a rapid deterioration in their performance^26,31–38^ and hence limits the material’s utility in most practical environments and where longer term operation is desired. Once the oxide layer forms on the surface of the material, it can only be dissolved by strong acids as TiO_2_ can withstand temperatures of over 450 ^∘^C^39^. Such acids however have the propensity of degrading the MXenes.

Given high current synthesis costs and the difficulties of scaling manufacturing towards industrial throughput^40^, the ability to prolong the shelf life of MXene is critical in ensuring their potential for translation into commercial reality. Such an effort is, nevertheless, hugely challenging. While oxidation can be prevented by storing the material in hermetically-sealed containers at 5 ^∘^C^26^, this does not address their stability during use when exposed to ambient environments. The only other efforts so far have been limited to improving the oxidative stability of MXenes by delaying their oxidation kinetics, either during initial synthesis of the material, or subsequently during storage^33,35–37,41^. Limbu et al., for example, showed that it is possible to suppress the oxidation of colloidal MXene nanosheets with the use of L-ascorbic acid^42^. Lee et al., on the other hand, demonstrated that MXene films annealed under hydrogen at high temperatures above 300 ^∘^C for extended periods (0.5–45 h) exhibited better oxidation resistance^25^. There has, however, yet to be a facile and rapid way in which oxidized MXenes can be recovered and hence restored after they have deteriorated.

In this work, we show, quite peculiarly, that exposing oxidized thick micron- or thin nanometer-order MXene films, and even those oxidized as flakes whilst dispersed in solution, to very brief (1 min) high frequency (10 MHz) nanometer-amplitude surface-localized electromechanical vibrations in the form of surface reflected bulk waves (SRBWs; 10 nm order amplitude surface and hybrid acoustic waves generated on a chipscale piezoelectric substrate^43^ that have found extraordinary utility for a myriad of applications, such as microfluidic actuation and manipulation^44–47^, and the synthesis of bulk and 2D crystals^48^, including that of transition metal dichalcogenides^49,50^ and MXenes^38,51^) leads to efficient removal of the oxide layer, therefore allowing their electrical and electrochemical performance to be recovered—under ambient conditions and without the need for chemical additives or reducing agents. Up to 61% reduction in their total oxide content, as determined through x-ray photoelectron spectroscopy (XPS), is observed, with concomitant recovery in the material’s capacitive performance to a level comparable to that for pristine films prior to oxidation.

## Results and discussion

**Fig. 1:**
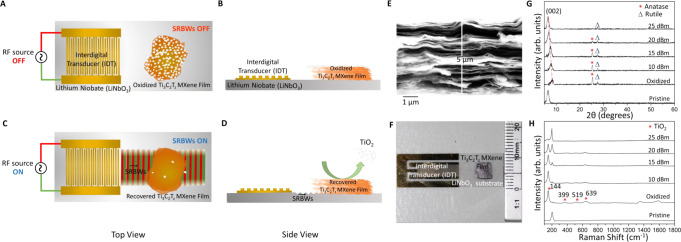
Experimental setup and materials characterization. **A**, **C** Top, and, (**B**, **D**) side view schematics of the experimental setup in which a 5 µm thick oxidized Ti_3_C_2_T_z_ MXene film (a scanning electron microscopy (SEM) image of which is shown in (**E**)) is placed atop the surface reflected bulk wave (SRBW) resonator, which comprises a chipscale single-crystal LiNBO_3_ piezoelectric substrate, shown in (**F**). In (**C**, **D**), the SRBW—generated by applying a sinusoidal electrical signal from a radio frequency (RF) source to interdigital transducer (IDT) electrodes photolithographically patterned onto the LiNbO_3_ substrate—propagates along and through the substrate and is transmitted into the Ti_3_C_2_T_z_ MXene film through a thin water coupling layer. In the control experiment (**A**, **B**), the SRBW is not excited. **G** Powder x-ray diffraction (XRD), and, (**H**) Raman spectra of the pristine, control (oxidized) and SRBW-irradiated films MXenes at different powers.

Exposing a 5 μm thick Ti_3_C_2_T_z_ MXene film, left to oxidize for a month under ambient conditions, to the SRBW—generated by applying a sinusoidal radio frequency (RF) signal to interdigital transducer (IDT) electrodes patterned on a single-crystal piezoelectric (lithium niobate; LiNbO_3_) substrate on which the film is placed (Fig. 1A–F)—at different intensities (10, 15, 20 and 25 dBm) over just one minute can be seen to induce chemical compositional changes to the material. The x-ray diffraction (XRD) spectra in Fig. 1G, for example, shows a gradual increase in the relative intensity of the (002) peak for Ti_3_C_2_T_z_ at a low diffraction angle of 8.4^∘^ with increasing SRBW power, and an accompanying decrease in the anatase [Joint Committee on Powder Diffraction Standards (JCPDS)^52^ no. 71-1167] and rutile [JCPDS no. 21-1276] TiO_2_ peaks at 25.3^∘^ and 27.5^∘^, respectively. This indicates the progressive removal of the oxide layer on the Ti_3_C_2_T_z_ MXene film^26,53^, with respect to the oxidized control film that was not subjected to the SRBW irradiation. Parenthetically, we also note that the (002) Ti_3_C_2_T_z_ peak shifts to lower 2*θ*, from 8.4^∘^ to 6.54^∘^, similar to that of the pristine film, thereby suggesting an approximate 3 Å expansion in the *c*-lattice parameter. This can be attributed to the adsorption of a layer of water molecules (water was used to couple the vibration from the substrate into the film) between the layers of the MXene film, consistent with that observed previously^29^. These observations are supported by the Raman spectroscopy results in Fig. 1H that also show a gradual decrease in the relative intensities of the characteristic peaks for anatase and rutile TiO_2_ at 144, 399, 519, and 639 cm^−1^ with increasing SRBW power, in addition to a concomitant increase in the vibrational mode at 202 cm^−1^ characteristic of Ti_3_C_2_T_z_ MXenes^26,54^.

**Fig. 2:**
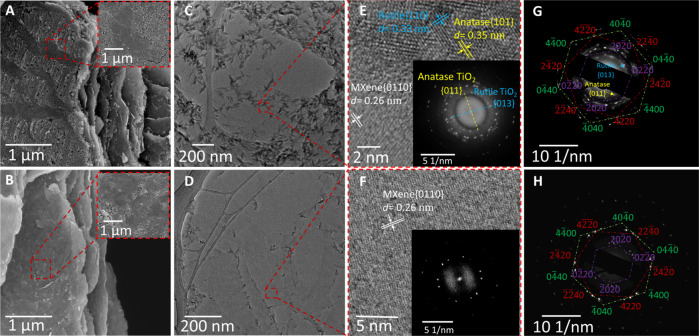
Electron microscopy. **A**, **B** Scanning electron microscopy (SEM), (**C**, **D**) transmission electron microscopy (TEM), (**E**, **F**) high resolution TEM (HRTEM) images, and, (**G**, **H**) corresponding selected area electron diffraction (SAED) patterns of the oxidized (control; top row: **A**, **C**, **E**, **G**) and SRBW-recovered (bottom row: **B**, **D**, **F**, **H**; 25 dBm) Ti_3_C_2_T_z_ MXene films. The insets in (**E**) and (**F**) show the corresponding Fast Fourier Transform (FFT) patterns, in which the anatase {101} and {013} TiO_2_ planes are apparent for the control (oxidized) film. The indexed diffraction spots exhibit the typical hexagonal diffraction pattern of Ti_3_C_2_T_z_.

Inspection of the films under scanning electron microscopy (SEM; Fig. 2A, B) and transmission electron microscopy (TEM; Fig. 2C, D), prior to (top row, Fig. 2) and following (bottom row, Fig. 2) exposure to the SRBW at the highest intensity (25 dBm) reveals a significant decrease in the coverage of the relatively uniform layer of TiO_2_ that initially coated the surface of the oxidized film (Fig. S1, Supplementary Information). The removal of the oxide layer with the SRBW irradiation is further confirmed by the contrast in the high-resolution TEM (HRTEM) images between the control (unexcited) and SRBW-recovered samples in Fig. 2E,F. Specifically, the HRTEM image for the control (oxidized) film in Fig. 2E shows the characteristic {0110} Ti_3_C_2_T_z_ plane, as well as a disorder in the lattice due to the presence of the surface oxide layer (visible from the diffuse centrosymmetric background in the Fast Fourier Transform (FFT) pattern in the inset of Fig. 2E). Further, we also note the 3.5 Å and 3.3 Å lattice spacings that respectively correspond to the {101} and {110} planes of the rhombic-like anatase and prismatic rutile phases of TiO_2_^33,35^; the {101} anatase TiO_2_ plane is further apparent from the selected area electron diffraction (SAED) pattern in Fig. 2G, in addition to the hexagonal diffraction pattern characteristic of Ti_3_C_2_T_z_ MXene. In contrast, the HRTEM image of the film subjected to the SRBW at the highest intensity, i.e., 25 dBm (Fig. 2F, H), appears to be relatively devoid of anatase and rutile TiO_2_, while retaining the characteristic Ti_3_C_2_T_z_ lattice spacing and diffraction pattern, therefore confirming the removal of a significant proportion of the oxidized layer from the film’s surface.

**Fig. 3:**
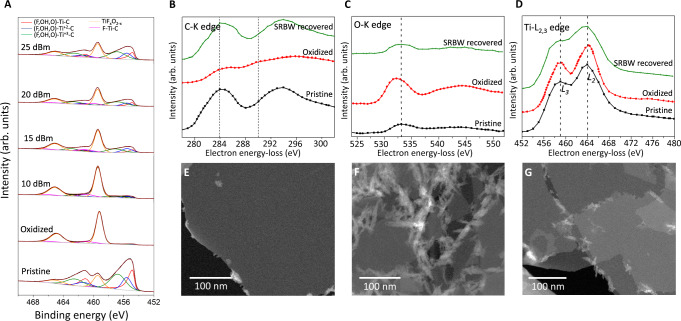
Chemical composition. **A** X-ray photoelectron spectroscopy (XPS) spectra associated with the Ti 2*p* region for the pristine, control (oxidized), and SRBW-recovered Ti_3_C_2_T_z_ films at increasing powers; the curves were fitted for the (F, OH, O)–Ti–C, (F, OH, O)–Ti^2+^–C, (F, OH, O)–Ti^3+^–C, TiF_x_O_2−x_, and F_z_–Ti–C species (Table S2, Supplementary Information). **B–D** Electron energy-loss spectroscopy (EELS) analysis (after background subtraction and plural scattering deconvolution) of the pristine, control (oxidized) and SRBW-recovered (25 dBm) films, with the oxidized control having **B** a C–K edge showing a diminished onset peak at 284 eV and a carboxide peak at 290 eV, **C** a −1 eV shift in the O–K edge, and, **D** +0.3 and +0.2 eV shifts in the Ti − L_2_ and Ti − L_3_ edges, respectively, all of which are recovered after SRBW exposure; the dashed lines indicate the relative shift in the peaks. High-angle annular dark-field scanning transmission electron microscopy (HAADF-STEM) scans for the (**E**) pristine, (**F**) control (oxidized), and, (**G**) SRBW-recovered (25 dBm) films.

The change in the areal coverage of the MXene assigned species [i.e., species belonging to Ti_3_C_2_T_z_: (F, OH, and/or O)–Ti–C, (F, OH, and/or O)–Ti^2+^–C, (F, OH, and/or O)–Ti^3+^–C, F_z_–Ti–C; and surface oxides: TiF_x_O_2−x_] as a function of the SRBW power can be seen in the deconvolved peaks (details of the peak deconvolution analysis are reported in Table S1 in the Supplementary Information) of the high-resolution XPS spectra associated with the Ti 2*p* region of MXene in Fig. 3A. In particular, we note a decrease in TiF_x_O_2−x_ from 79% in the control (oxidized) Ti_3_C_2_T_z_ film, consistent with typical oxidation values between 40% and 80% previously reported in the literature^33,35–37,41^, to 73%, 52%, 47%, and 31%, respectively, following SRBW irradiation at 10, 15, 20, and 25 dBm. This further corroborates our earlier XRD and HRTEM results indicating significant reduction in the TiO_2_ content in the film—by up to approximately 61% at the highest SRBW intensity, in comparison to the oxidized control sample (79%) (Table S2, Supplementary Information), which is also confirmed from the chemical formulas of the samples calculated through the XPS analysis (Table S3, Supplementary Information).

The presence of the initial oxide layer on the film, as well as its removal by the SRBW, can additionally be verified through an elemental analysis of the control and SRBW-irradiated samples using electron energy-loss spectroscopy (EELS; Fig. 3B–D). Given that Ti is neither deposited or desorbed, we assume the number of Ti atoms in the layer to remain constant, and hence normalize the edge intensities against that for the Ti − L_2,3_ edge (Fig. 3D). Determination of the O to Ti stoichiometric ratio, denoted as x, from the O–K (Fig. 3C) and Ti − L_2,3_ (Fig. 3D) edge spectra then shows that the total O amount (x = 2.52; Table S4, Supplementary Information) in the control (oxidized) film, is significantly reduced upon exposure of the film to the SRBW at 25 dBm (x = 1.22; Table S4, Supplementary Information), such that it approaches that of the pristine sample (x = 0.69; Table S4, Supplementary Information), due to the reduction of TiO_2_ species upon irradiation. This can also be seen from further analysis of the XPS spectra in the C 1*s*, O 1*s* and the F 1*s* regions (Fig. S2 and Table S5, Supplementary Information), in which we observe no significant change in the surface termination groups between the pristine and SRBW-restored films, as summarized in Table S6 in the Supplementary Information.

Examination of the C–K edge spectra (Fig. 3B), on the other hand, shows a diminished onset peak at 284 eV that is shifted relative to the SRBW-irradiated sample by +0.95 eV and +1.06 eV for the control (oxidized) and pristine films, respectively. A structural change in the control film can also be observed, in which part of the intensity of the peak is shifted toward a shoulder centered at approximately 290 eV, which can be attributed to carboxide (C_y_O_z_) formation^35^. Accompanying shifts in the O–K, Ti − L_2_ and Ti − L_3_ peaks of −1, +0.3 and +0.2 eV, respectively, are also evident in the oxidized control (Fig. 3B,D). These combined energy shifts in the oxidized control sample indicate charge transfer from Ti and C to O, which can be attributed to the higher electronegativity of O as a consequence of the formation of additional Ti–O and new C–O bonds, as well as a reduction in Ti–C bonds, all of which are commensurate with oxidation of the film^35^. Moreover, the increase in total O content of the oxidized film can also be attributed to the formation of TiO_2_ and the oxidation of C atoms at the Ti-vacancies to form amorphous carbon aggregates^33^. Importantly, we note the reversal of these oxidation-induced structural changes in the spectra following exposure of the oxidized MXene film to the SRBWs at 25 dBm, in which the onset peak associated with the C–K edge at 284 eV is clearly prominent, as are the disappearances of the carboxide shoulder at 290 eV, and the shifts in the O–K and Ti − L_2,3_ peaks, all of which indicate the removal of Ti–O bonds in the irradiated sample.

The high-angle annular dark-field scanning transmission electron microscopy (HAADF-STEM) images in Fig. 3E–G also indicate significant removal of TiO_2_ upon irradiation of the film with the SRBW. During its oxidation, the surface of the pristine Ti_3_C_2_T_z_ MXene film (Fig. 3E) is typically covered with TiO_2_, which manifests as a uniformly distributed layer of nanoparticles on the surface of the control (oxidized) film (Fig. 3F), whose stability on the film’s surface is governed by van der Waals (approximately −3 eV) and electrostatic repulsion (approximately +0.1 eV) forces; the former (i.e., the attractive force) being dominant at pH values between 1.6 and 7.0^55–57^. Upon exposure of the film to the SRBW, we postulate that the evanescent electric field associated with the SRBW nonlinear electromechanical coupling^48,58^ leads to dipole polarization of the TiO_2_ layer (similar dipole induction with the SRBW has been observed in other crystalline materials^59^). This, in turn, increases the electrostatic repulsion, which, together with the unprecedentedly large 10^8^ m s^−2^ (i.e., 10 million *g*’s) surface acceleration associated with the SRBW^43^, results in the ejection of the layer of TiO_2_ nanoparticles from the surface of the film (Fig. 3G). While it is possible that the SRBW treatment could also potentially result in desorption of the oxygen functional groups from the Ti sites within the MXene structure, we were not able to either prove or disprove this given our inability to quantify the surface terminations in the control (oxidized) sample due to the difficulty in distinguishing between the MXene’s surface terminations and the –O and –OH groups adsorbed on TiO_2_ since they have the same binding energy.

As seen from the contact angle measurements in Fig. S3 (Supplementary Information), the removal of the surface oxide layer of the Ti_3_C_2_T_z_ film following SRBW exposure can also be seen to restore the film to its original wettability state. Due to the abundance of oxide particles on the surface of the oxidized film, we note that the control sample yielded contact angles of approximately 60^∘^ ± 3. 1^∘^, similar to that previously reported^60^. Upon irradiation of the film with the SRBW at 25 dBm, the contact angle was found to increase to approximately 68^∘^ ± 3. 6^∘^, close to that of the pristine Ti_3_C_2_T_z_ film (approximately 72^∘^ ± 2. 8^∘^) and similar to reported values for Ti_3_C_2_T_z_ MXenes with low amounts of surface TiO_2_^31,61^.

**Fig. 4:**
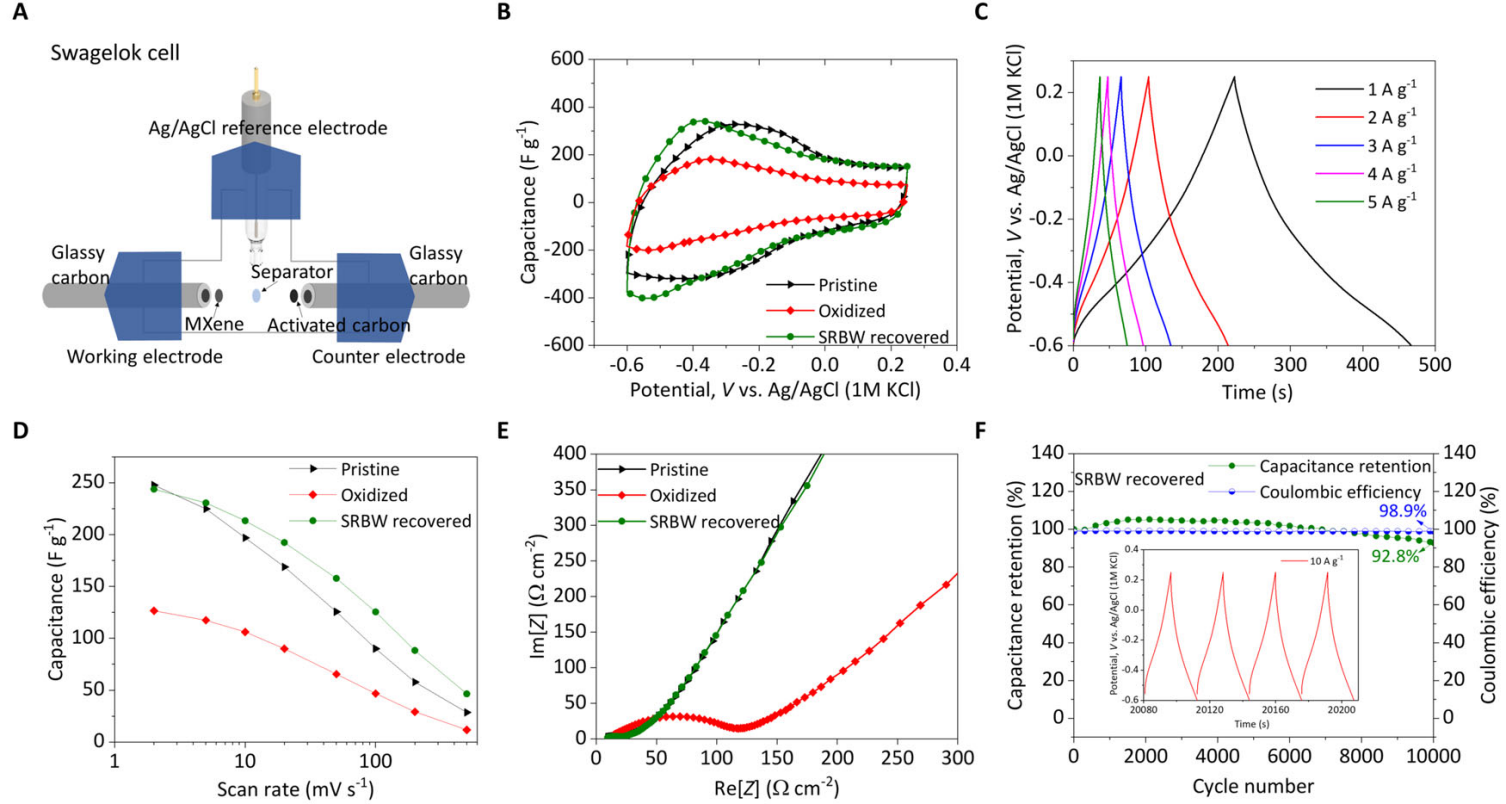
Electrochemical performance. **A** Schematic depiction of the three-electrode cell setup with the Ti_3_C_2_T_z_ MXene film as the working electrode, activated carbon as the counter electrode, and Ag/AgCl immersed in 1 M KCl as the reference electrode. Glassy carbon was used as the current collector for both the working and counter electrodes, which were partitioned using separators to prevent contact. **B** Cyclic voltammetry (CV) behavior of the as-prepared pristine (black), oxidized control (blue), and SRBW-restored (red) Ti_3_C_2_T_z_ MXene film electrodes at a scan rate of 10 mV s^−1^. **C** Charge–discharge analysis (galvanostatic cycling with potential limitation, GCPL) of the SRBW-restored electrode film at different current densities. **D** Gravimetric capacitance of the pristine and SRBW-restored Ti_3_C_2_T_z_ MXene film electrodes for different scan rates. **E** Nyquist plots for the pristine, control (oxidized) and SRBW-restored Ti_3_C_2_T_z_ MXene electrodes at open circuit potentials; Re[*Z*] and Im[*Z*] denote the real and imaginary impedances of the system. **F** Life cycle performance of the SRBW-restored Ti_3_C_2_T_z_ MXene film at 500 mV s^−1^ showing 92.8% capacity retention and 98.9% Coulombic efficiency; the inset shows results from the GCPL stability test performed at 10 A g^−1^ over 10,000 cycles. In all of the cases, the SRBW power is held constant at 25 dBm.

In addition to restoring the electrical conductivity of the film from almost negligible levels in the oxidized film back to a similar level (approximately 97%) of its pristine counterpart (Fig. S4, Supplementary Information), we additionally probe the ability of the SRBW-induced surface modification of the Ti_3_C_2_T_z_ MXene film to restore its capacitive performance through a series of electrochemical tests using a conventional three-electrode setup in 1 M H_2_SO_4_ electrolyte, in which the pristine (unoxidized; as prepared), control (oxidized) and SRBW-treated (restored; 25 dBm) films were employed as the working electrodes (Fig. 4A). Typical of the pseudocapacitive behavior of Ti_3_C_2_T_z_ MXenes^53^, no clearly-separated oxidation and reduction peaks were observed in the cyclic voltammetry (CV) curves in Fig. 4B (see also Fig. S4, Supplementary Information) for each of these films, indicating their high electrochemical reversibility^62^. This observation is notable for the oxidized control, suggesting that oxidation does not alter the characteristic electrochemical behavior of MXene.

Nevertheless, we note a marked decrease in the electrochemical performance of the oxidized film, with a gravimetric capacitance for the control sample calculated to be 127 F g^−1^ at a scan rate of 2 mV s^−1^ (Fig. S5, Supplementary Information)—48.2% lower than that for the pristine film prior to oxidation (245 F g^−1^; Fig. S5, Supplementary Information); the degradation in performance being a consequence of the high TiO_2_ surface content that reduces Ti–C bonding and the film’s conductivity, which, in turn, diminishes the overall charge transfer^63,64^. Removal of the surface oxides upon irradiation of the film with the SRBW at 25 dBm, in contrast, can be seen to restore the gravimetric capacitance of the material to around 98% of the value of the pristine electrode (242 F g^−1^ at a scan rate of 2 mV s^−1^; Fig. 4B); a small overpotential difference of approximately −0.1 V can be observed, likely due to the additional overall electrode resistance arising from residual TiO_2_ particles on the SRBW-treated film (noting the difference in the TiO_2_ content between the SRBW-treated film (31%) and the pristine film (17%) in comparison with that in the control (oxidized) sample (79%)). Charge–discharge analysis of the SRBW-treated electrode under galvanostatic cycling with potential limitation (GCPL) at different current densities (Fig. 4C), on the other hand, confirms that the restored electrode retains highly symmetrical charge–discharge profiles with linear deviations characteristic of the pseudocapacitive behavior of Ti_3_C_2_T_z_ MXene in H_2_SO_4_ aqueous electrolyte^33,51,53^.

Rate performance testing of the pristine and SRBW-restored films further verifies that both electrodes possessed similar gravimetric capacitances (Fig. 4D), and that the long-term stability of the SRBW-treated electrode was shown to be capable of maintaining up to 92.8% of its initial capacitance after 10,000 cycles at a specific current of 10 A g^−1^, with a Coulombic efficiency of approximately 98.9% for the same film (Fig. 4F); we note the decline in capacitance at higher scan rates to be common for pseudocapacitive electrodes given that the rate at which the electrochemical reaction occurs is controlled by electrolyte ion diffusion at high scan rates^65^ (Fig. 4D). In contrast to the control (oxidized) sample, the high frequency region in the Nyquist plots in Fig. 4E reveal low charge-transfer resistances for the pristine and SRBW-recovered electrodes, as evident by the suppression of the characteristic semicircular arc profile, with higher slopes associated with ion diffusion in the low-frequency range that suggest an increased rate of electrolyte transport. These again further confirm the capability of the SRBW treatment to restore the oxidized Ti_3_C_2_T_z_ MXene film back to a state that is close to its pristine counterpart^53^.

Multiple SRBW restoration treatments following its re-oxidation are also possible without detriment to the material’s integrity. This can be seen from the results in Fig. S6 in the Supplementary Information following three successive oxidation and SRBW-treatment cycles, wherein we additionally demonstrate that the treatment also works regardless of the way by which the sample is oxidized (dispersed in solution, as opposed to being oxidized as a film; in addition, the second and third oxidation steps were also carried out differently by heating the material at 100 ^∘^C for 12 h). Further, we show that the technique is equally applicable to thin flakes (15 nm thickness; Fig. S7 in the Supplementary Information) of a different MXene composition (in this case, Mo_2_CT_z_) to similar effect (approximately 64% reduction in total oxide content; Fig. S8, Supplementary Information), with comparable surface terminations between the pristine and SRBW-recovered samples (Tables S7 and S8, Supplementary Information).

Despite their vast potential, the use of MXenes in practical applications is plagued by its tendency to oxidize under ambient environments, resulting in a marked deterioration of its performance and therefore severely limiting its operational lifespan. While there have been attempts to prevent oxidation or to improve its stability, the possibility for reversing its oxidation state and hence restoring the performance of the material has, to our best knowledge, not been reported to date. In this work, we demonstrate the possibility of exploiting high frequency MHz-order electromechanical vibrations in the form of SRBWs to remove up to around 61% of the oxides from the surface of exposed 5 µm thick Ti_3_C_2_T_z_ MXene films, and around 64% from that of 15 nm thick Mo_2_CT_z_ flakes, under ambient conditions, without requiring chemical additives or reducing agents. While the phenomenon itself is unexpected, it is perhaps not entirely surprising given that the SRBWs, while having only nanometer amplitudes, possess surface accelerations that are on the order of 10^8^ m s^−2^^43^—adequately sufficient to eject the oxide layer off the surface, although leaving the MXene structure unaltered. We show that the reversibility in the film’s oxidative state with the SRBW treatment allows the electrochemical performance of the material to be restored close to the level of its pristine (unoxidized) state. The treated film, for example, is seen to retain its characteristic pseudocapacitive behavior, and its gravimetric capacitance is observed to be restored to around 98% of that of its pristine value, with good capacitive retention (approximately 93%) after 10,000 cycles.

Since the same SRBW device has been demonstrated for the synthesis of MXene films and quantum dots^38,51^, we foresee the possibility of a single platform for both MXene synthesis and restoration. Moreover, given the short treatment duration (1 min), the platform’s chipscale dimension, and the possibility for fabricating the SRBW devices at low costs down to approximately US$1/device by exploiting the economies-of-scale associated with mass manufacture, we also anticipate the potential for scaling the technology through massive parallelization for high throughput processing as a practical means for extending the lifetime of MXene films in practical industrial settings. Such potential for rejuvenating spent or degraded material for reuse is envisaged to address the high current costs of MXene synthesis, in addition to rendering its use more economically and sustainably viable, therefore overcoming the present technological and market barriers faced for its commercial adoption.

## Methods

### SRBW device

The SRBW device, depicted in Fig. 1A–D and shown in Fig. 1F, comprises a 500 μm thick single-crystal piezoelectric 128^∘^ Y-rotated, X-propagating lithium niobate (LiNbO_3_) chipscale substrate (Roditi Ltd., London, UK) on which 30 pairs of parallel interleaved interdigital transducer (IDT) fingers with an aperture of 3.9 mm are patterned by sputter coating 10 nm and 200 nm thick layers of titanium and gold, followed by standard photolithography and wet etching. The SRBW is generated by applying a sinusoidal electrical signal with applied voltages of 1.5, 3, 6, and 9 V_rms_ (corresponding to powers of 10, 15, 20, and 25 dBm, respectively) at the resonant frequency *f* to the IDT via a signal generator (SML01, Rhode & Schwarz GmbH & Co. KG, Munich, Germany) and RF amplifier (ZHL-5W-1+, Mini-Circuits, Brooklyn, NY, USA). *f* (here, 10 MHz) is measured using a vector network analyzer (ZNB4, Rhode & Schwarz GmbH & Co. KG, Munich, Germany), and is determined by the gap and width of the IDT fingers to correspond to a quarter wavelength *λ*/4 = *c*/4*f* ≈ 100 µm, in which *c* denotes the sound phase speed in LiNbO_3_.

### Ti_3_C_2_T_z_ synthesis

Ti_3_AlC_2_ MAX phase was synthesized through a solid–liquid reaction between Ti (325 mesh, 99.999 wt.% purity; Alfa Aesar, Kandel, Germany), TiC (325 mesh, 99.5 wt.% purity; Alfa Aesar, Kandel, Germany), and Al (325 mesh, 99.5 wt.% purity; Alfa Aesar, Kandel, Germany) at a molar ratio of 1:2:1, respectively. The powders were first mixed in an agate mortar, following which they were placed in an alumina crucible and heated in a horizontal tube under a 5 sccm Ar flow at a rate of 5 ^∘^C min^−1^ to 1450 ^∘^C, at which temperature it was held for 120 min. The sample was then cooled, crushed using a mortar and pestle, and sieved through a 450 mesh. Multilayer Ti_3_C_2_ was then synthesized by slowly immersing 1 g of the Ti_3_AlC_2_ powder (450 mesh) in a polytetrafluoroethylene (PTFE) bottle containing 20 ml of 12 M hydrochloric acid (HCl, technical grade; Fisher Scientific GTF AB, Göteborg, Sweden) and 2.3 M lithium fluoride (LiF, 98+%; Alfa Aesar, Kandel, Germany). The mixture was subsequently agitated using a PTFE-coated magnetic stirrer bar for 24 h at 35 ^∘^C in an oil bath, and washed with 40 ml of 1 M HCl followed by three further washing cycles in 40 ml of 1 M lithium chloride (LiCl, 98+%; Alfa Aesar, Kandel, Germany). The multilayered MXene was next thrice washed with 40 ml of N_2_-deaerated deionized (DI) water till a dark supernatant is observed. In each washing cycle, 40 ml DI water was added to the multilayer powder in a centrifuge tube, hand-shaken for 1 min, and centrifuged at 4200 ×*g* for 1 min, from which the supernatant was decanted. After washing, the MXene mixture was further agitated by hand for 5 min followed by centrifugation at 2058 ×*g* for 1 h. The supernatant that was obtained, which contained single to few layer Ti_3_C_2_T_z_ flakes in water, was of a concentration between 1–4 mg ml^−1^. A flexible freestanding Ti_3_C_2_T_z_ film was then produced by vacuum filtration of 15 ml (1 mg/ml) concentrated colloidal solution through a nanopolypropylene membrane (3501 Coated PP, 0.064 µm pore size, Celgard LLC, Charlotte, NC). The 34 mm diameter and 5 µm thick filtered film (Fig. S1, Supplementary Information) was finally dried in air overnight and kept in a glove box under N_2_ until required.

### Mo_2_CT_z_ synthesis

The detailed method for the synthesis of Mo_2_CT_z_ MXenes from Mo_2_Ga_2_C can be found elsewhere^66^. Briefly, Mo_2_Ga_2_C was produced by a solid–liquid reaction between Mo_2_C and Ga, wherein Mo_2_C powder (99.5 wt.%, 2 *μ*m; Alfa Aesar, Kandel, Germany) was mixed with Ga pellets (99.999 wt.%; Wuhan Xinrong New Material Co. Ltd., Wuhan, China) with a mass ratio of 6:10, respectively, and placed in an alumina crucible that was subsequently heated in a horizontal quartz furnace under a 5 sccm Ar flow at a rate of 10 ^∘^C min^−1^ to 800 ^∘^C, at which temperature it was held for 72 h. After cooling the furnace, the lightly sintered product was crushed using a mortar and pestle, and slowly added to 12 M hydrochloric acid (HCl; Sigma Aldrich AB, Stockholm, Sweden) at a ratio of 1 g to 10 ml and left overnight to remove any excess Ga. The mixture was then washed with DI water until it reached pH ≈ 6. Next, 1 g of Mo_2_Ga_2_C (particle size < 35 µm) was slowly added to 20 ml of 25 wt.% hydrofluoric acid (HF; Sigma Aldrich AB, Stockholm, Sweden) in a PTFE bottle and the mixture placed in an oil bath on a hot plate at 55 ^∘^C under agitation using a PTFE-coated magnetic stirrer for 160 h. After completion of the reaction, the mixture was washed with N_2_-deaerated DI water over several cycles, each with volume 40 ml, until pH ≈ 6 was reached. To delaminate the resultant multilayer Mo_2_CT_z_ MXene, 10 ml of 40 wt.% tetrabutylammonium hydroxide (TBAOH; Sigma Aldrich AB, Stockholm, Sweden) in water was first mixed with the multilayered Mo_2_CT_z_ powder and shaken for 5 min in a vortex mixer (LSE, Corning Inc., Glendale, AZ) at a speed of 162 ×*g*, following which the TBAOH was decanted by centrifuging the mixture at 6048 ×*g* for 1 min. The sedimented powder was then thrice washed each with 40 ml of N_2_-deaerated DR water; care was taken to avoid shaking during this step to prevent spontaneous delamination. Finally, delamination was achieved by adding 40 ml N_2_-deaerated DI water and agitating the mixture for 10 min in the vortex mixer at 162 ×*g*, before centrifuging for 1 h at 1050 ×*g* to produce a colloidal suspension of single Mo_2_CT_z_ MXene flakes. To produce the Mo_2_CT_z_ film, a glass substrate coated with indium tin oxide on one side was cleaned and fastened to the rotating plate of a spin coater. 30 µl of the delaminated Mo_2_CT_z_ colloidal solution (≈ 15 mg ml^−1^ concentration) was then drop casted on the substrate using a pipette and the substrate spun coated at 112 ×*g* for 30 s.

### Oxidation procedure

For the control and SRBW-irradiation experiments, films were left to oxidize in air under ambient conditions for one month. Alternatively, for the experiments demonstrating multiple successive SRBW treatment cycles, a Ti_3_C_2_T_z_ MXene solution in DI water was delaminated through the aforementioned method and subsequently oxidized partially in an oven at 100 ^∘^C for 12 h. Next, a freestanding film was constructed from the solution using the technique described above, following which it was treated with the SRBW. The SRBW-recovered film was then bath sonicated in 10 ml of DI water until it formed a suspension, which was re-oxidized under the same conditions and filtered to form a film, prior to exposure to the SRBW again for the next recovery cycle.

### SRBW irradiation of the MXene film

The 34 mm diameter freestanding Ti_3_C_2_T_z_ MXene film was cut into 0.5 cm × 0.5 cm × 5 µm pieces, and either used as prepared in its pristine form, or oxidized as per the procedures described above. The oxidized film was subsequently placed atop the SRBW substrate with an intervening 5 µl water layer to couple the SRBW into the film. The film was then irradiated with the SRBW at different powers (10, 15, 20, and 25 dBm) for 1 min at approximately 18 ± 3 °C (Fig. 1B). A control sample was also included in which the oxidized film was not subjected to the SRBW irradiation. For subsequent characterization, the film was then used as is, or crushed using a mortar and pestle.

### Characterization

#### X-ray diffraction (XRD)

Powder XRD was conducted to determine the structure of the films using a powder diffractometer (D8 Advance, Bruker Pty. Ltd., Preston, VIC, Australia) under Cu K*α* radiation at 35 mA and 40 kV (*λ* = 1.54 Å) over a 2*θ* range of 5^∘^–60^∘^ with a step size of 0.016^∘^ and scan rate of 1.2 min^−1^.

#### Raman spectroscopy

Raman scattering spectra of the films on pure silicon substrates were acquired across 100–4000 cm^−1^ at approximately 18 ± 3 °C using a microspectrometer (LabRAM HR Evolution, Horiba Advanced Techno Co. Ltd., Kyoto, Japan) with an Ar ion laser (532 nm) at 0.5 mW power and a grating with 1800 lines mm^−1^. Magnification during the experiment was set to 100×.

#### Atomic force microscopy (AFM)

The thickness of the Mo_2_CT_z_ MXene flakes were measured through imaging using an atomic force microscope (AFM, Dimension Icon; Bruker Corp., Billerica, MA, USA) and analysed with the supplied software (NanoScope Analysis 2.0; Bruker Corp., Billerica, MA, USA).

#### Scanning electron microscopy (SEM)

The morphology and thickness of the MXene films were determined from high-resolution field-emission SEM (Nova NanoSEM 450, FEI, Hillsboro, OR) imaging under a 3 kV electron beam by attaching them onto substrates with carbon conductive tape.

#### Transmission electron microscopy (TEM)

Low magnification TEM (JEM-1010, JEOL Ltd., Akishima, Tokyo, Japan) and high-resolution field-emission TEM (HRTEM; JEM-200F, JEOL Ltd., Akishima, Tokyo, Japan) imaging of the MXene films was conducted at operating voltages of 100 kV and 200 kV, respectively. The samples were prepared by grinding the MXene films using a mortar and pestle, and depositing a suspension of the obtained flakes in isopropanol onto holey carbon-coated copper grids. The following parameters were employed: spot size = 1, *α* = 1, condenser aperture number 3 (CA3), selected area aperture number 2 (SA2).

#### High-angle annular dark-field scanning transmission electron microscopy (HAADF-STEM)

HAADF-STEM (HRTEM; JEM-200F, JEOL Ltd., Akishima, Tokyo, Japan) images were captured at an operating voltage of 100 kV. A beam current of 0.1 nA was applied to protect the sample during the imaging process. All images were low-pass filtered for better visibility. An inverse fast Fourier transform was applied to enhance the images.

#### Electron energy-loss spectroscopy (EELS)

Compositional analysis was performed with dual-EELS using the same sample and holder combination in the field-emission TEM operating in STEM mode at the same operating voltage and beam current. A spectrometer (GIF Continuum S, Gatan Inc., Pleasanton, CA) was used to collect core loss C–K, O–K and Ti − L_2,3_ edge spectra, and the intensities were normalized with respect to the Ti peak. The composition was determined by averaging across the spectra within the area of interest and using the built-in functions in the analysis software that was supplied (DigitalMicrograph, Gatan Inc., Pleasanton, CA), with uncertainty in the stoichiometry calculated from the variance formula using the ratio of C and Ti.

#### X-ray photoelectron spectroscopy (XPS)

XPS data for the MXene films mounted on a silicon substrate were collected using a photoelectron spectrometer (AXIS Supra, Kratos Analytical Ltd., Manchester, UK) with a monochromated Al K*α* x-ray source (1486.7 eV) under high vacuum. The analyzer pass energy used for all cases was 20 eV with a step size of 0.050 eV. The binding energy (BE) scale, on the other hand, was referenced to the Fermi-edge (E_F_), which was set to a BE of 0 eV. Peak fitting was carried out using CasaXPS (Version 2.3.24 PR 1.0; Casa Software Ltd., Teignmouth, UK) as in refs. 67 and 68 for Ti_3_C_2_T_z_ and ref. 69 for Mo_2_CT_z_.

#### Electrochemical measurements

Cyclic voltammetry (CV), galvanostatic cycling with potential limitation (GCPL) and electrochemical impendence spectroscopy (EIS) techniques were used to investigate the differences in the electrochemical performance of pristine (unoxidized), control (oxidized) and SRBW-restored Ti_3_C_2_T_z_ electrodes. All electrochemical measurements were performed in the open at approximately 18 ± 3 °C and 55% ± 5% humidity without a climatic/environmental chamber using a VSP potentiostat (BioLogic, Seyssinet-Pariset, France) in a three-electrode plastic Swagelok® cell (Swagelok Eastern Australia, Melbourne, VIC, Australia), in which Ti_3_C_2_T_z_ MXene discs (3 mm diameter; 5 ± 0.15 µm average thickness, calculated from seven different measurements of its cross-sectional area; 55 µg mass) served as the working electrodes. The activated carbon counter electrode was prepared by mixing 95 wt.% of the material (YP-50 AC, Kuraray Co. Ltd., Tokyo, Japan) with 5 wt.% PTFE binder (60 wt.% dispersion in water) (Sigma-Aldrich Corp., St. Louis, MO, USA). The mixture was then rolled, dried and punched into 6 mm diameter and 150 µm thick discs with a weight of approximately 1 mg. An Ag/AgCl electrode immersed in 1 M KCl was utilized as the reference electrode, and glassy carbon electrodes were used as current collectors. The upper and lower potentials (−0.6 to +0.20 V) were determined according to the protocol used in ref. 11. The electrodes’ gravimetric capacitance *C*_*g*_, determined from CV profiles at scan rates *SR* from 2 to 500 mV s^−1^, is given by

1 $${C}_{g}=\frac{1}{m{{\Delta }}V}\int\nolimits_{{V}_{1}}^{{V}_{2}}\frac{1}{SR}\,dV,$$

in which *m* denotes the electrode mass and Δ*V* the voltage window. EIS was performed at the open circuit potential (OCP) at an amplitude of 10 mV and across frequencies ranging from 10 MHz and 100 kHz. GCPL tests were performed at 1, 2, 3, 5, and 10 A g^−1^, and stability measurements were obtained at 10 A g^−1^ for 10,000 cycles.

#### Current–voltage measurements

The current-voltage (*I*–*V*) characteristics of the film were measured with an electrometer (4200–SCS, Keithley Instruments Inc., Cleveland, OH, USA) and a manual probe station at approximately 18 ± 3 °C across a voltage range from −1 to 1 V. Briefly, the 0.5 cm × 0.5 cm × 5 µm pristine, control (oxidized) and SRBW-recovered films were placed atop two gold pads (1 µm separation) patterned on the surface of the LiNbO_3_ substrate away from the IDTs, for contact with the probes.

#### Contact angle measurement

Static contact angles of sessile DI water drops (30 µl), carefully placed atop dried pristine, control (oxidized) and SRBW-restored MXene films, were measured at approximately 18 ± 3 ^∘^C by analyzing their images, acquired with a camera (UI-1220LE-M-GL, IDS Imaging Development Systems GmbH, Obersulm, Germany) following equilibriation of the drop after 10 s, using Surface Meter^TM^ software (Ningbo NB Scientific Instruments Co. Ltd., Ningbo, China). The average value and standard error of the contact angle was obtained from five separate measurements.

## Supplementary information

Supporting Information

Peer Review File

## Data Availability

The datasets generated during this study have been deposited in the Figshare database under accession code 21344394 (https://figshare.com/articles/dataset/Source_Data_xlsx/21344394).
