# Supplementary material for: Recovery of oxidized two-dimensional MXenes through high frequency nanoscale electromechanical vibration
Source: Nat Commun. 2023 Jan 3;14:3. doi: 10.1038/s41467-022-34699-3 (PMC9810719; doi:10.1038/s41467-022-34699-3)
Supplement: Supplementary file 1 — Supporting Information [file 41467_2022_34699_MOESM1_ESM.pdf]

Supplementary Information File

Recovery of Oxidized Two-Dimensional MXenes Through High Frequency Nanoscale Electromechanical Vibration

Heba Ahmed,[1] Hossein Alijani,[1] Ahmed El-Ghazaly,[2] Joseph Halim,[2]
Billy J. Murdoch,[3] Yemima Ehrnst,[1] Emily Massahud,[1] Amgad R. Rezk,[1] Johanna Rosen[2] &
Leslie Y. Yeo[1]

[1] *Micro/Nanophysics Research Laboratory, School of Engineering, RMIT University, Melbourne, VIC 3000, Australia*
[2] *Materials Design Division, Department of Physics, Chemistry, and Biology (IFM), Linköping University, Linköping SE-58183, Sweden*
[3] *RMIT Microscopy and Microanalysis Facility, RMIT University, Melbourne, VIC 3000, Australia*

Email: leslie.yeo@rmit.edu.au, amgad.rezk@rmit.edu.au

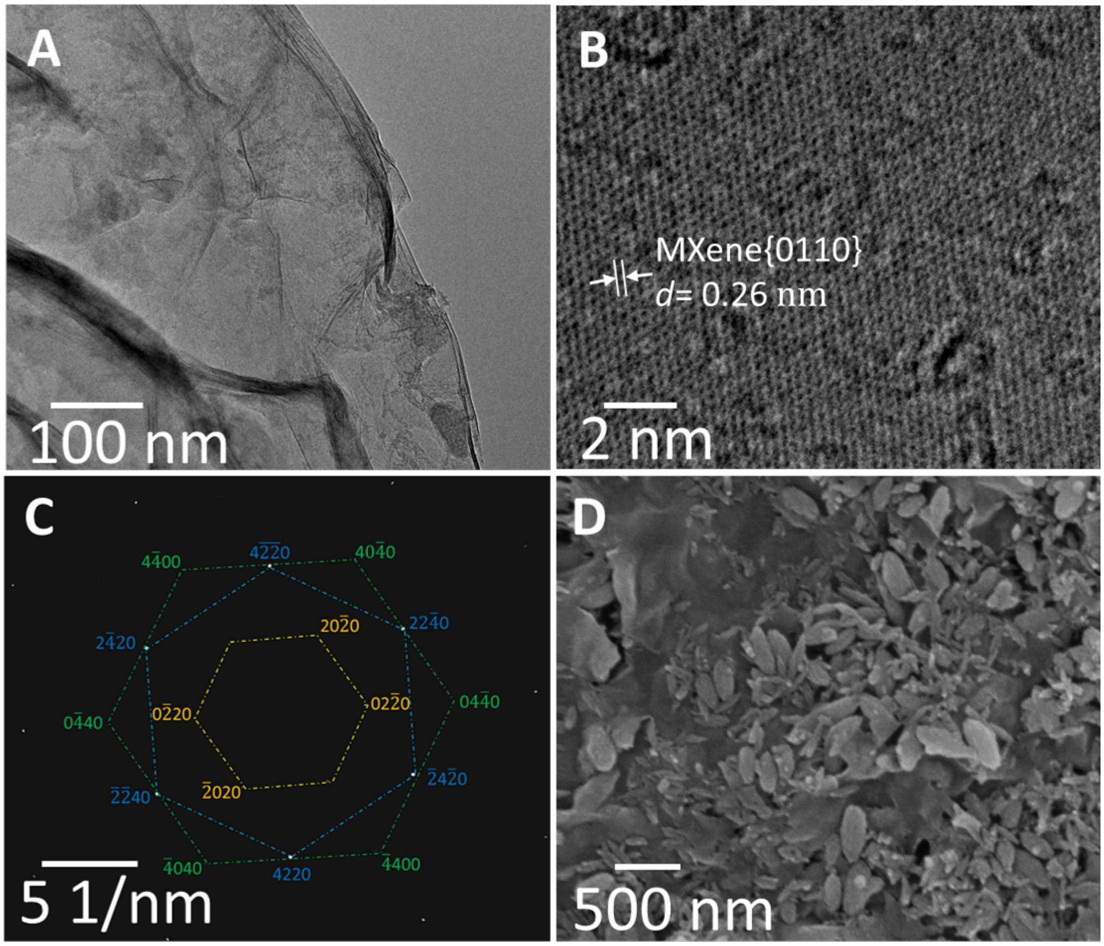

Fig. S1. MXene characterization. (A) Transmission electron microscopy (TEM), and, (**B**) high-resolution TEM images of pristine (as-prepared) $Ti_3C_2T_z$ MXene prior to their oxidation. The 0.26 nm d-spacing shown in (B) is characteristic of the {0110} basal plane of $Ti_3C_2T_z$. (C) Selected area electron diffraction (SAED) pattern of the pristine sample, characteristic of $Ti_3C_2T_z$ and showing no signs of oxidation. (**D**) Scanning electron microscopy (SEM) image of the oxidized (control) MXene film, showing spindle- and spherically-shaped TiO_2 on its surface.

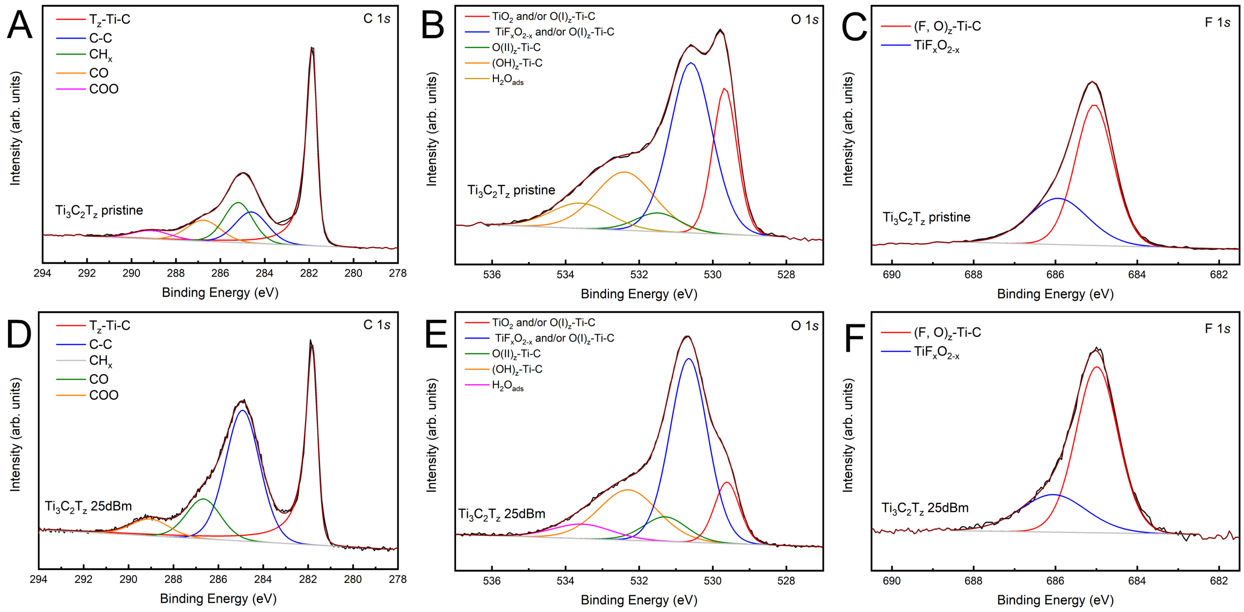

Fig. S2. $Ti_3C_2T_z$ MXene surface characterization. High-resolution x-ray photoelectron spectra (XPS) for (**A**,**B**,**C**) pristine (as prepared, prior to their oxidation), and, (**D**,**E**,**F**) SRBW-restored (25 dBm) $Ti_3C_2T_z$ MXene films for the (**A**,**D**) C 1*s*, (**B**,**E**) O 1*s*, and, (**C**,**F**) F 1*s* regions.

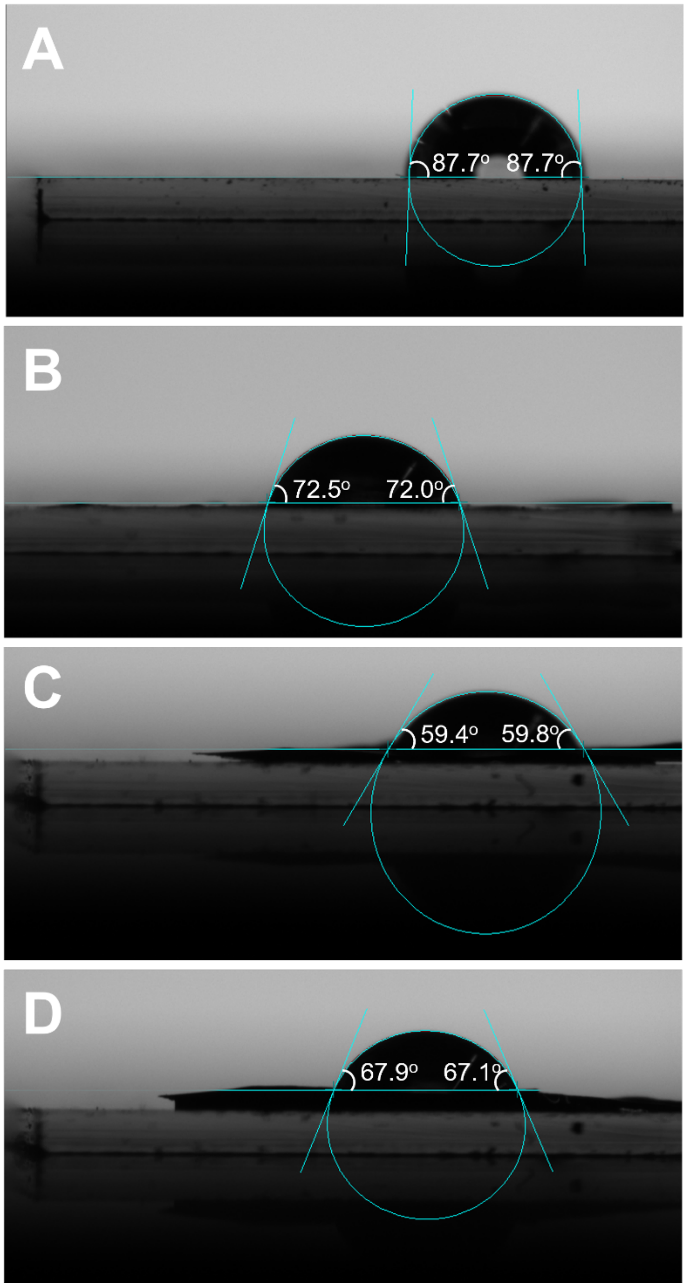

Fig. S3. Wettability measurements. Contact angles θ of a sessile deionized (DI) water drop placed atop (**A**) the surface reflected bulk wave (SRBW) device comprising a single-crystal piezoelectric lithium niobate ($LiNbO_3$) substrate ($\theta \approx 90^{\circ} \pm 2.2^{\circ}$), (**B**) the pristine (as prepared, prior to their oxidation) $Ti_3C_2T_z$ MXene film ($\theta \approx 72^{\circ} \pm 2.8^{\circ}$), (**C**) the control (oxidized) $Ti_3C_2T_z$ MXene film prior to SRBW exposure ($\theta \approx 60^{\circ} \pm 3.1^{\circ}$), and, (**D**) the SRBW-restored (25 dBm) $Ti_3C_2T_z$ MXene film ($\theta \approx 68^{\circ} \pm 3.6^{\circ}$). The average value and standard error of the contact angles quoted were obtained from five separate measurements.

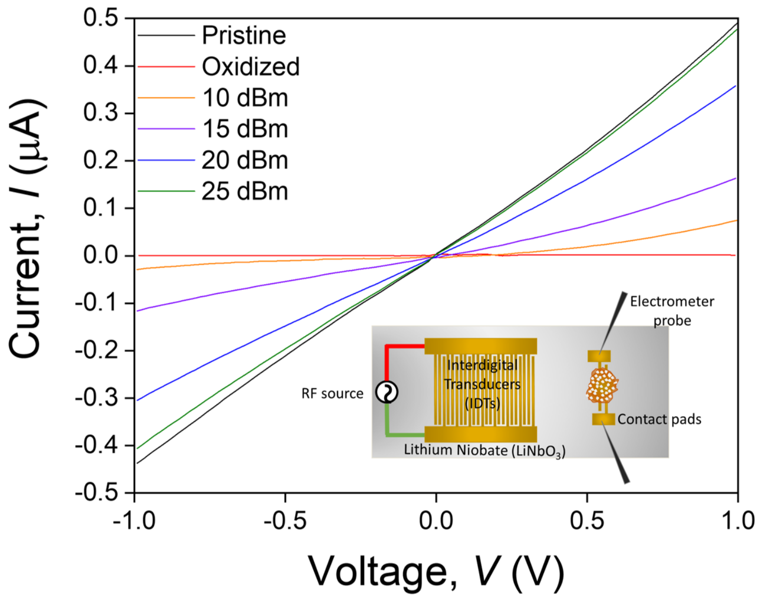

Fig. S4. Electrical conductivity. Current–voltage (*I*–*V*) measurements of the pristine (as prepared, prior to their oxidation) and control (oxidized) $Ti_3C_2T_z$ MXene films, and those restored through SRBW exposure at increasing powers. The inset shows a schematic representation of the electrometer probe setup used, in which the films were placed atop two gold contact pads (1 μm separation) patterned atop the SRBW substrate, which comprised a single-crystal piezoelectric lithium niobate ($LiNbO_3$) substrate on which an interdigitated transducer (IDT) was patterned to drive the SRBW. It is noteworthy that the conductivity curves for both the pristine and SRBW-restored films show a slight nonlinear behavior, which could arise from surface defects, or vacancies (Deng et al., *Adv Opt Mater* **7**, 1801521, 2019).

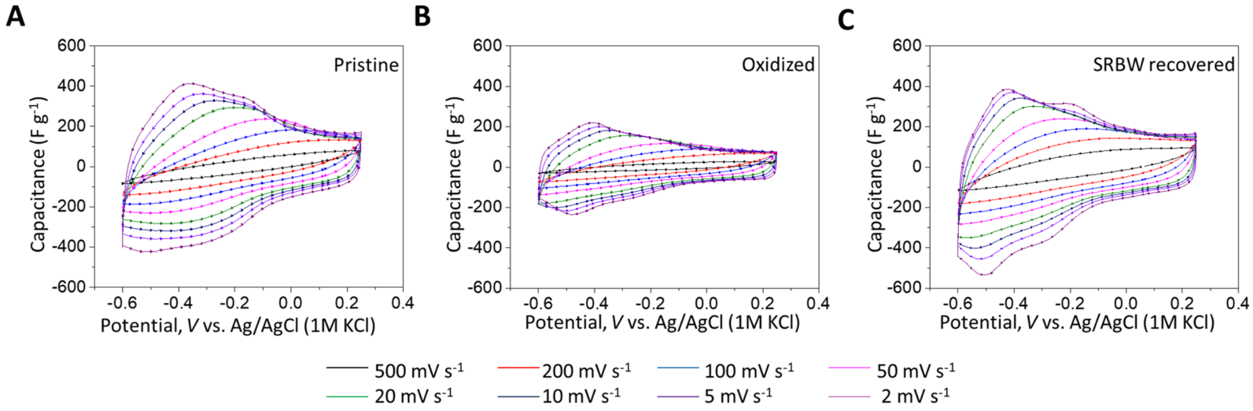

Fig. S5. Electrochemical performance. Cyclic voltammograms for (**A**) pristine (as prepared, prior to their oxidation), (**B**) control (oxidized), and, (**C**) SRBW-restored (25 dBm) $Ti_3C_2T_z$ MXene electrodes at scan rates of 2, 5, 10, 20, 50, 100, 200 and 500 mV s^{-1} in 1 M H_2SO_4 with a three-electrode configuration.

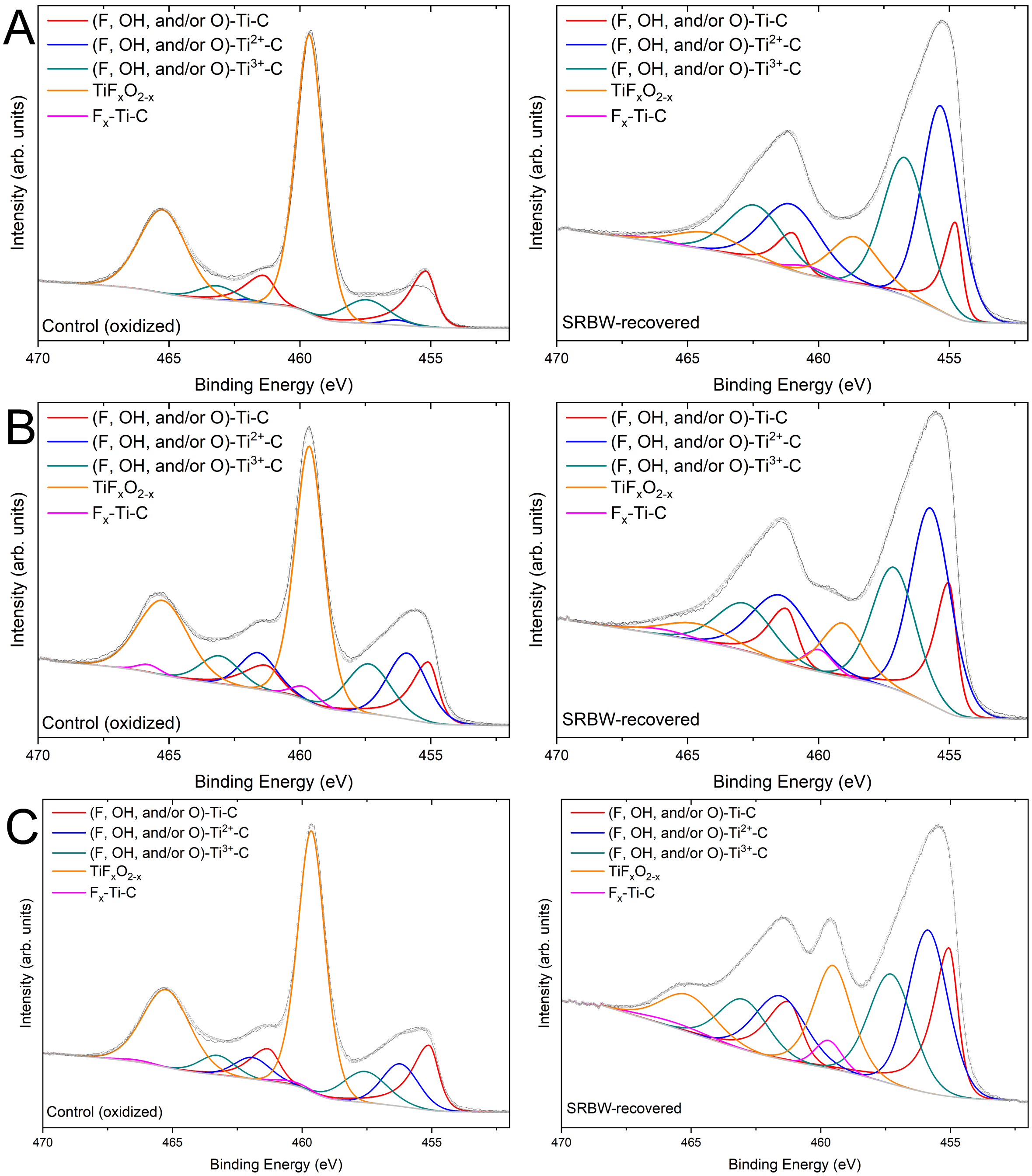

Fig. S6. $Ti_3C_2T_z$ MXene surface characterization for repeated SRBW recovery treatments. High resolution XPS spectra in the Ti 2*p* region for $Ti_3C_2T_z$ MXene following (**A**) one, (**B**) two, and, (**C**) three successive oxidation (left column; control) and SRBW recovery (right column) cycles. To demonstrate the applicability of the recovery technique for samples oxidized in different ways, we conducted the first oxidation step by exposing the sample to air under ambient conditions, and the second and third oxidation steps under heat by placing the sample on a hotplate at 200 ^{o}C for 12 h. For this experiment, the $Ti_3C_2T_z$ flakes were originally dispersed and subsequently oxidized in solution.

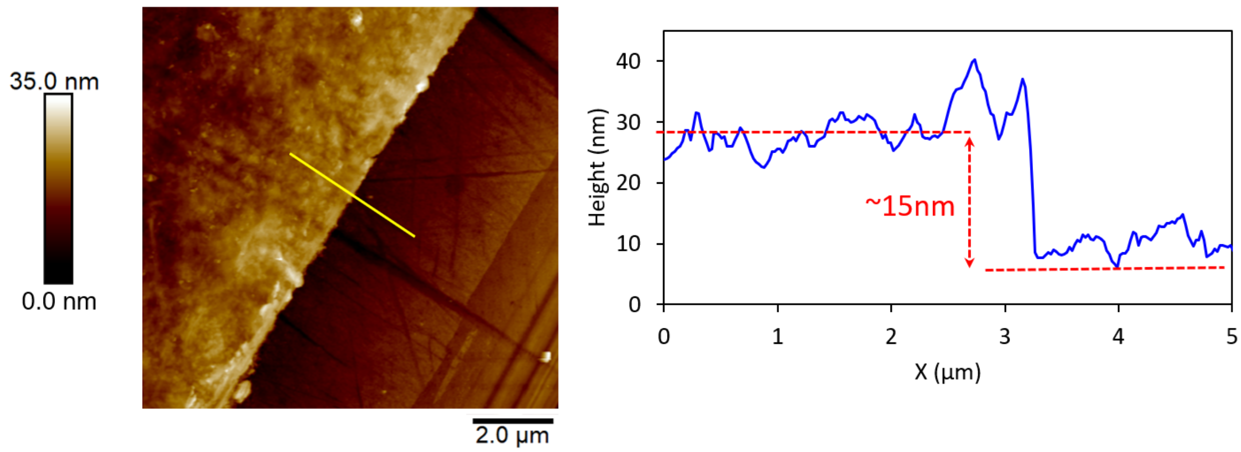

Fig. S7. Film thickness measurement. Atomic force microscopy (AFM) image of a thin Mo_2CT_z MXene film showing a thickness (also indicated by the colour bar for the image) of approximately 15 nm as demonstrated from the line scan measurement.

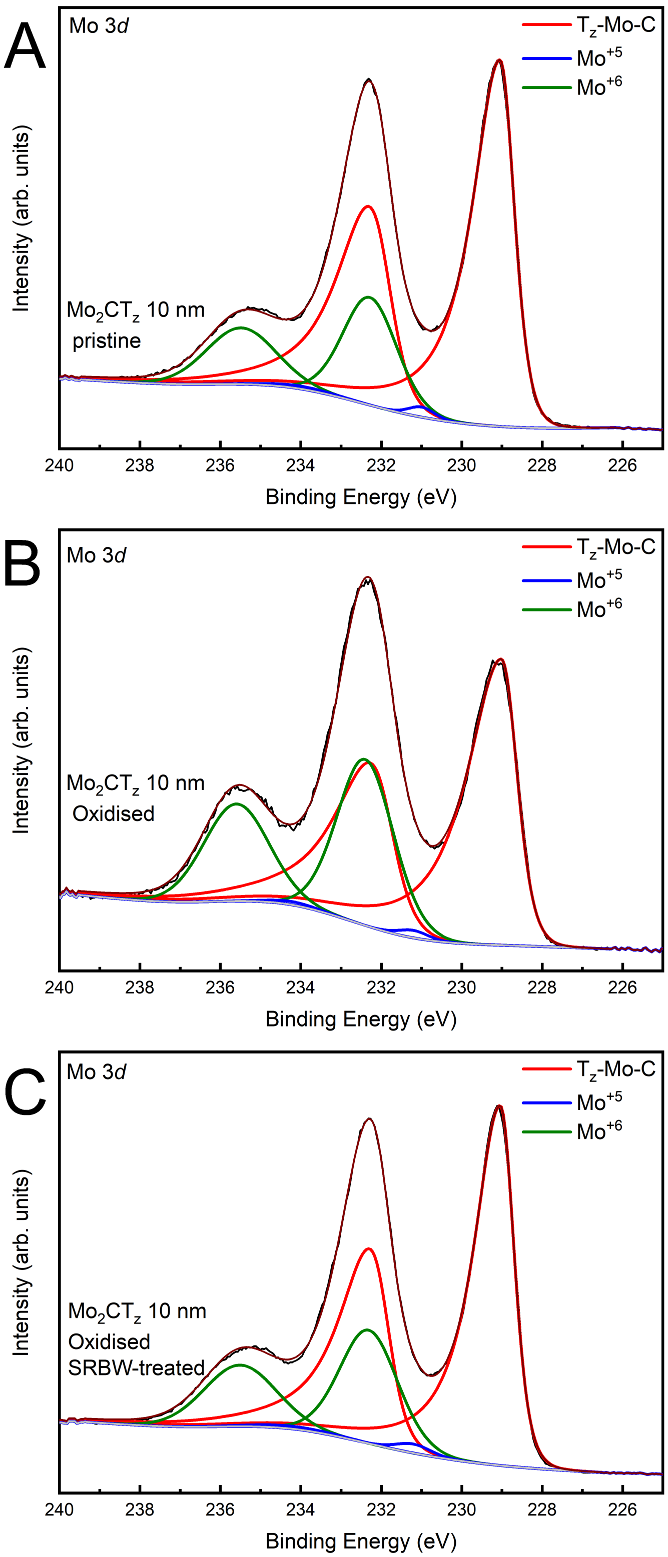

Fig. S8. Mo_2CT_z MXene surface characterization. High-resolution XPS spectra in the Mo 3*d* region for 15 nm (**A**) pristine (as prepared, prior to their oxidation), (**B**) control (oxidized), and, (**C**) SRBW-restored (25 dBm) Mo_2CT_z MXene films.

Table S1. Atomic composition. Global atomic composition of the pristine (as prepared, prior to their oxidation), control (oxidized) and SRBW-restored $Ti_3C_2T_z$ MXene films, obtained from x-ray photoelectron spectroscopy (XPS).

Elements	Ti %(at.)	C %(at.)	O %(at.)	F %(at.)	Cl %(at.)	Al %(at.)
**$Ti_3C_2T_z$ (pristine; as prepared)**	24.4±0.1	41.5±0.5	18.7±0.2	12.4±0.2	3.0±0.4	< 0.1
**$Ti_3C_2T_z$ (control; oxidized)**	14.8 ± 0.3	37.7 ± 0.7	39.1 ± 0.6	7.9 ± 0.4	0.5 ± 0.3	< 0.1
**$Ti_3C_2T_z$ (10 dBm)**	14.2 ± 0.3	43.5 ± 0.8	35.0 ± 0.7	6.2 ± 0.4	1.1 ± 0.3	< 0.1
**$Ti_3C_2T_z$ (15 dBm)**	15.1 ± 0.4	45.6 ± 1.0	31.5 ± 0.7	6.8 ± 0.4	1.0 ± 0.4	< 0.1
**$Ti_3C_2T_z$ (20 dBm)**	14.4 ± 0.4	49.4 ± 0.9	28.4 ± 0.7	5.7 ± 0.4	2.1 ± 0.3	< 0.1
**$Ti_3C_2T_z$ (25 dBm)**	24.1 ± 0.5	37.1 ± 1.0	27.5 ± 0.9	8.5 ± 0.6	2.8 ± 0.4	< 0.1

Table S2. Chemical composition. Percentage of surface oxyfluorides (TiF_xO_{2-x}) obtained from XPS peak analysis of the Ti 2*p* region of the pristine (as prepared, prior to their oxidation), control (oxidized) and SRBW-restored $Ti_3C_2T_z$ MXene films.

Sample	% TiF_xO_{2-x}
**$Ti_3C_2T_z$ (pristine; as prepared)**	17
**$Ti_3C_2T_z$ (control; oxidized)**	79
**$Ti_3C_2T_z$ (10 dBm)**	73
**$Ti_3C_2T_z$ (15 dBm)**	52
**$Ti_3C_2T_z$ (20 dBm)**	47
**$Ti_3C_2T_z$ (25 dBm)**	31

Table S3. XPS peak fitting. XPS peak fitting results in the Ti 2*p* region for the pristine (as prepared, prior to their oxidation), control (oxidized) and SRBW-restored $Ti_3C_2T_z$ MXene films (Halim et al., *Appl Surf Sci* **362**, 406, 2016; Halim et al. *RSC Adv* **8**, 36785, 2018).

Sample	Binding energy (eV)[a]	Full Width Half Maximum (eV)[a]	Fraction	Assigned to
**$Ti_3C_2T_z$ (pristine; as prepared)**	454.7 (460.9)	0.6 (1.2)	0.26	(F, OH, and/or O)-Ti-C
	455.4 (461.1)	1.4 (2.1)	0.19	(F, OH, and/or O)-Ti^{2+} -C
	456.8 (462.5)	2.4 (2.5)	0.37	(F, OH, and/or O)-Ti^{3+}-C
	459.4 (465.1)	1.14(2.5)	0.17	TiF_xO_{2-x}
	460.5 (466.3)	1.2 (3.0)	0.01	F_z-Ti-C
**$Ti_3C_2T_z$ (control; oxidized)**	454.6 (460.8)	0.7 (1.4)	0.03	(F, OH, and/or O)–Ti–C
	455.2 (460.9)	1.2 (1.9)	0.01	(F, OH, and/or O)–Ti^{2+}–C
	456.8 (462.4)	2.6 (2.0)	0.06	(F, OH, and/or O)–Ti^{3+}–C
	459.2 (464.9)	1.1 (2.0)	0.79	TiF_xO_{2-x}
	460.7 (466.2)	2.5 (2.5)	0.14	F_z–Ti–C
**$Ti_3C_2T_z$ (10 dBm)**	454.7 (460.9)	0.4 (1.0)	0.02	(F, OH, and/or O)–Ti–C
	455.4 (461.1)	1.3 (2.0)	0.04	(F, OH, and/or O)–Ti^{2+}–C
	457.0 (462.7)	2.6 (1.7)	0.10	(F, OH, and/or O)–Ti^{3+}–C
	459.4 (465.1)	1.1 (2.0)	0.73	TiF_xO_{2-x}
	460.9 (466.3)	2.2 (3.0)	0.11	Fz–Ti–C
**$Ti_3C_2T_z$ (15 dBm)**	454.8 (461.0)	0.5 (1.0)	0.06	(F, OH, and/or O)–Ti–C
	455.6 (461.3)	1.6 (2.0)	0.13	(F, OH, and/or O)–Ti^{2+}–C
	457.2 (462.9)	2.5 (2.1)	0.19	(F, OH, and/or O)–Ti^{3+}–C
	459.4 (465.1)	1.1 (2.0)	0.52	TiF_xO_{2-x}
	461.0 (466.5)	2.4 (3.0)	0.10	F_z–Ti–C
**$Ti_3C_2T_z$ (20 dBm)**	454.8 (461.0)	0.5 (1.0)	0.10	(F, OH, and/or O)–Ti–C
	455.6 (461.3)	1.6 (2.0)	0.11	(F, OH, and/or O)–Ti^{2+}–C
	457.2 (462.9)	2.5 (2.1)	0.27	(F, OH, and/or O)–Ti^{3+}–C
	459.4 (465.1)	1.1 (2.0)	0.47	TiF_xO_{2-x}
	461.0 (466.5)	2.0 (3.0)	0.05	F_z–Ti–C
**$Ti_3C_2T_z$ (25 dBm)**	454.8 (461.0)	0.6 (1.0)	0.15	(F, OH, and/or O)–Ti–C
	455.6 (461.3)	1.6 (2.0)	0.17	(F, OH, and/or O)–Ti^{2+}–C
	457.2 (462.9)	2.5 (2.1)	0.34	(F, OH, and/or O)–Ti^{3+}–C
	459.4 (465.1)	1.2 (2.1)	0.31	TiF_xO_{2-x}
	460.4 (466.2)	2.5 (2.7)	0.03	F_z–Ti–C

[a] Values in parentheses correspond to the $2p_{1/2}$ component.

Table S4. Compositional analysis. Electron energy-loss spectroscopy (EELS) analysis of the pristine (as prepared, prior to their oxidation), control (oxidized), and SRBW-restored $Ti_3C_2T_z$ MXene films. The O and C compositions were determined relative to Ti.

Sample	Element Shell Signal	Composition %(at.)	Relative Composition	Areal Denisty (atm/nm^2)	Volume Density (atm/ nm^3)
**$Ti_3C_2T_z$ (pristine; as prepared)**	**C–K**	50 ± 2	1.70	410 ± 20	23.9 ± 1.2
	**Ti–$L_{2,3}$**	29.4 ± 1.2	1.00	240 ± 20	14.0± 1.4
	**O–K**	20.5 ± 0.8	0.69	169 ± 9	9.8 ± 0.5
**$Ti_3C_2T_z$ (control; oxidized)**	**C–K**	41.4 ± 1.4	2.49	311 ± 16	19.0 ± 0.9
	**Ti–$L_{2,3}$**	16.6 ± 0.6	1.00	125 ± 12	7.6 ± 0.8
	**O–K**	42.0 ± 1.4	2.52	315 ± 16	19.2 ± 1.0
**SRBW-restored $Ti_3C_2T_z$**	**C–K**	41.9 ± 1.5	1.60	339 ± 17	19.8 ± 1.0
	**Ti–$L_{2,3}$**	26.2 ± 1.0	1.00	210 ± 20	12.4 ± 1.2
	**O–K**	31.9 ± 1.2	1.21	258 ± 13	15.1 ± 0.8

Table S5. $Ti_3C_2T_z$ MXene XPS peak fitting. XPS peak fitting results in the Ti 2*p*, C 1*s*, O 1*s*, and F 1*s* regions for the pristine (as prepared, prior to their oxidation) and SRBW-restored $Ti_3C_2T_z$ MXene films at 25 dBm (Halim et al., *Appl Surf Sci* **362**, 406, 2016; *RSC Adv* **8**, 36785, 2018).

Region	Sample	BE (eV)[a]	Full Width Half Maximum (eV)[a]	Fraction	Assigned to
Ti 2*p*	$Ti_3C_2T_z$ (pristine; as prepared)	454.7 (460.9)	0.6 (1.2)	0.26	(F, OH, and/or O) –Ti–C
		455.4 (461.1)	1.4 (2.1)	0.19	(F, OH, and/or O) –Ti^{2+}–C
		456.8 (462.5)	2.4 (2.5)	0.37	(F, OH, and/or O)–Ti^{3+}–C
		459.4 (465.1)	1.14(2.5)	0.17	TiF_xO_{2-x}
		460.5 (466.3)	1.2 (3.0)	0.01	F_x–Ti–C
	$Ti_3C_2T_z$ (25 dBm)	454.8 (461.0)	0.6 (1.0)	0.15	(F, OH, and/or O)–Ti–C
		455.6 (461.3)	1.6 (2.0)	0.17	(F, OH, and/or O)–Ti^{2+}–C
		457.2 (462.9)	2.5 (2.1)	0.34	(F, OH, and/or O)–Ti^{3+}–C
		459.4 (465.1)	1.2 (2.1)	0.31	TiF_xO_{2-x}
		460.4 (466.2)	2.5 (2.7)	0.03	F_x–Ti–C
C 1*s*	$Ti_3C_2T_z$ (pristine; as prepared)	281.8	0.5	0.45	T_z–Ti–C
		284.6	1.7	0.23	C–C
		285.2	1.5	0.09	CH_x
		286.8	1.8	0.16	C–O
		289.1	2	0.07	COO
	$Ti_3C_2T_z$ (25 dBm)	281.8	0.6	0.36	T_z–Ti–C
		284.9	1.8	0.44	C–C
		286.7	1.8	0.13	C–O
		289	2	0.07	COO
O 1*s*	$Ti_3C_2T_z$ (pristine; as prepared)	529.7	0.8	0.22	TiO_2 and O(I)$_z$–Ti–C
		530.6	1.3	0.43	TiF_xO_{2-x} and O(I)$_z$–Ti–C
		531.5	1.4	0.05	O(II)$_z$–Ti–C/OR[b]
		532.4	1.9	0.21	(OH)$_z$–Ti–C/OR[b]
		533.6	1.9	0.09	H_2O_{ads}/OR[b].
	$Ti_3C_2T_z$ (25 dBm)	529.6	0.8	0.12	TiO_2 and O(I)$_z$–Ti–C
		530.7	1.2	0.52	TiF_xO_{2-x} and O(I)$_z$–Ti–C
		531.3	1.4	0.08	O(II)$_z$–Ti–C/OR[b]
		532.3	1.9	0.22	(OH)$_z$–Ti–C/OR[b]
		533.6	1.9	0.06	H_2O_{ads}/OR[b].
F 1*s*	$Ti_3C_2T_z$ (pristine; as prepared)	685.1	1.1	0.66	(F, O)$_z$–Ti-C
		685.9	1.7	0.33	TiO_2F_{2-x}
	$Ti_3C_2T_z$ (25 dBm)	685	1.2	0.74	(F, O)$_z$–Ti–C
		686	1.9	0.26	TiO_2F_{2-x}

[a] Values in parentheses correspond to the $2p_{1/2}$ component.
[b] OR denotes organic compounds attached to the MXene sheets during intercalation with tetrabutylammonium hydroxide (TBAOH) and/or due to exposure of the samples to the ambient environment.

Table S6. Surface termination of $Ti_3C_2T_z$ MXene films. Quantification of the surface termination for the pristine (as prepared, prior to their oxidation) and SRBW-restored $Ti_3C_2T_z$ MXene films based on XPS peak fitting.

Sample	–O	–OH	–F	H_2O
**$Ti_3C_2T_z$ (pristine; as prepared)**	0.4	0.5	1.2	0.3
**$Ti_3C_2T_z$ (25 dBm)**	0.4	0.45	1.1	0.1

Table S7. Mo_2CT_z MXene XPS peak fitting. XPS peak fitting results in the Mo 3*d*, C 1*s*, O 1*s* and F 1*s* regions for the 15 nm pristine (as prepared, prior to their oxidation), control (oxidized) and SRBW-restored Mo_2CT_z MXene films (Zheng et al., *J Power Sources* **525**, 231064, 2022).

Region	Sample	Binding energy (eV)	Full Width Half Maximum (eV)	Fraction	Assigned to	Reference
Mo 3*d* [a]	**Mo_2CT_z (pristine; as prepared)**	229.0 (232.2)<br>231.0(234.0)<br>232.3 (235.5)	0.9 (1.2)<br>0.8 (1.9)<br>1.6 (2.0)	0.76<br>0.01<br>0.23	T_z–Mo–C<br>Mo^{+5}<br>Mo^{+6}	[1]<br>231.2 (234.3) [2, 3]<br>232.6 (235.7) [2, 3]
	**Mo_2CT_z (control; oxidized)**	229.0 (232.2)<br>231.2 (234.2)<br>232.4 (235.6)	0.9 (1.3)<br>0.9 (1.4)<br>1.7 (1.9)	0.65<br>0.01<br>0.34	T_z–Mo–C<br>Mo^{+5}<br>Mo^{+6}	[4]<br>231.2 (234.3) [2, 3]<br>232.6 (235.7) [2, 3]
	**Mo_2CT_z (25 dBm)**	229.0 (232.2)<br>231.2 (234.2)<br>232.3 (235.5)	0.8 (1.1)<br>1.0 (2.4)<br>1.7 (2.1)	0.72<br>0.01<br>0.27	T_z–Mo–C<br>Mo^{+5}<br>Mo^{+6}	[4]<br>231.2 (234.3) [2, 3]<br>232.6 (235.7) [2, 3]
C 1*s*	**Mo_2CT_z (control; oxidized)**	282.8<br>284.9<br>285.3<br>286.5<br>287.4<br>289.0	1.0<br>1.8<br>1.7<br>1.4<br>2.0<br>1.8	0.11<br>0.02<br>0.65<br>0.18<br>0.02<br>0.02	T_z–Mo–C<br>C-C<br>CH_x<br>C–O<br>C–F<br>COO	[1]<br>[5-7]<br>[5-7]<br>[5-7]<br>[8]<br>[5-7]
	**Mo_2CT_z (25 dBm)**	282.8<br>284.9<br>285.3<br>286.6<br>287.8<br>289.5	0.9<br>1.1<br>1.9<br>1.2<br>1.8<br>1.5	0.10<br>0.06<br>0.69<br>0.09<br>0.05<br>0.01	T_z–Mo–C<br>C–C<br>CH_x<br>C–O<br>C–F<br>COO	[3]<br>[5-7]<br>[5-7]<br>[5-7]<br>[8]<br>[5-7]
O 1*s*	**Mo_2CT_z (control; oxidized)**	530.0<br>531.0<br>532.1<br>533.6	1.3<br>1.1<br>1.9<br>2.0	0.49<br>0.15<br>0.31<br>0.05	Mo oxides<br>O–Mo–C<br>(OH)–Mo–C<br>and/or OR[b]<br>H_2O_{ads}<br>and/or OR[b]	530.3 ± 0.4 eV [2, 3]<br>[1]<br>[1]<br>[1]
	**Mo_2CT_z (25 dBm)**	530.1<br>531.0<br>532.1<br>534.1	1.3<br>1.3<br>2.0<br>2.1	0.47<br>0.22<br>0.28<br>0.03	Mo oxides<br>O–Mo–C<br>(OH)–Mo-C<br>and/or OR[b]<br>H_2O_{ads}<br>and/or OR[b]	530.3 ± 0.4 eV [2, 3]<br>[3]<br>[3]
F 1*s*	**Mo_2CT_z (control; oxidized)**	684.2<br>689.1	1.8<br>2.0	0.40<br>0.60	F–Mo–C<br>C-F	[2]<br>[8]
	**Mo_2CT_z (25 dBm)**	684.3<br>688.6	1.7<br>2.6	0.58<br>0.42	F–Mo-C<br>C–F	[3]<br>[8]

[a] Values in parenthesis correspond to $3d_{5/2}$ component. Areal ratios of $3d_{5/2}$ and $3d_{3/2}$ were constrained to 3:2.
[b] OR denotes organic compounds attached to the MXene sheets during intercalation with tetrabutylammonium hydroxide (TBAOH) and/or due to exposure of the samples to the ambient environment.

Table S8. Surface termination of Mo_2CT_z MXene. Quantification of the surface termination for the 15 nm pristine (as prepared, prior to their oxidation) and SRBW-restored Mo_2CT_z MXene based on XPS peak fitting.

**Sample**	**–O**	**–OH**	**–F**	**H_2O**
**Mo_2CT_z (pristine; as prepared)**	0.5	1.0	0.2	0.2
**Mo_2CT_z (25 dBm)**	0.8	0.7	0.2	0.1

References

[1] H. Lind, J. Halim, S.I. Simak, J. Rosen, Investigation of vacancy-ordered $Mo_{1.33}C$ MXene from first principles and x-ray photoelectron spectroscopy. *Physical Review Materials* 1 (2017) 044002.

[2] F. Werfel, E. Minni, Photoemission study of the electronic structure of Mo and Mo oxides. *Journal of Physics C: Solid State Physics* 16 (1983) 6091-6100.

[3] K. Arata, M. Hino, Solid catalyst treated with anion: XVIII. Benzoylation of toluene with benzoyl chloride and benzoic anhydride catalysed by solid superacid of sulfate-supported alumina. *Applied Catalysis* 59 (1990) 197-204.

[4] J. Halim, S. Kota, M.R. Lukatskaya, M. Naguib, M.-Q. Zhao, E.J. Moon, J. Pitock, J. Nanda, S.J. May, Y. Gogotsi, M.W. Barsoum, Synthesis and characterization of 2D molybdenum carbide (MXene). *Advanced Functional Materials* 26 (2016) 3118-3127.

[5] A. Proctor, P.M.A. Sherwood, X-ray photoelectron spectroscopic studies of carbon fibre surfaces. III—Industrially treated fibres and the effect of heat and exposure to oxygen. S*urface and Interface Analysis* 4 (1982) 212-219.

[6] E. Desimoni, G.I. Casella, A. Morone, A.M. Salvi, XPS determination of oxygen-containing functional groups on carbon-fibre surfaces and the cleaning of these surfaces. *Surface and Interface Analysis* 15 (1990) 627-634.

[7] Y. Xie, P.M.A. Sherwood, X-ray photoelectron spectroscopic studies of carbon fiber surfaces. Part 10. Valence-band studies interpreted by X-.alpha. calculations and the differences between PAN- and pitch-based fibers. *Chemistry of Materials* 1 (1989) 427-432.

[8] T. Nakajima, Y. Matsuo, B. žemva, A. Jesih, Synthesis of fluorine-graphite intercalation compounds by elemental fluorine and high oxidation-state transition-metal fluorides. *Carbon* 34 (1996) 1595-1598.
