# Supplementary material for: Recovery of oxidized two-dimensional MXenes through high frequency nanoscale electromechanical vibration
Source: Nat Commun. 2023 Jan 3;14:3. doi: 10.1038/s41467-022-34699-3 (PMC9810719; doi:10.1038/s41467-022-34699-3)
Supplement: Supplementary file 2 — Peer Review File [file 41467_2022_34699_MOESM2_ESM.pdf]

nature portfolio

Peer Review File

Recovery of Oxidized Two-Dimensional MXenes Through High Frequency Nanoscale Electromechanical Vibration

Open Access This file is licensed under a Creative Commons Attribution 4.0 International License, which permits use, sharing, adaptation, distribution and reproduction in any medium or format, as long as you give appropriate credit to the original author(s) and the source, provide a link to the Creative Commons license, and indicate if changes were made. In the cases where the authors are anonymous, such as is the case for the reports of anonymous peer reviewers, author attribution should be to 'Anonymous Referee' followed by a clear attribution to the source work. The images or other third party material in this file are included in the article's Creative Commons license, unless indicated otherwise in a credit line to the material. If material is not included in the article's Creative Commons license and your intended use is not permitted by statutory regulation or exceeds the permitted use, you will need to obtain permission directly from the copyright holder. To view a copy of this license, visit http://creativecommons.org/licenses/by/4.0/.

Reviewer comments, first round review:

Reviewer #1 (Remarks to the Author):

Title: Recovery of Oxidized Two-Dimensional Titanium Carbide Ti3C2Tz MXene Films Through High Frequency Nanoscale Electromechanical Vibration
Authors have proposed a new strategy in recovering oxidized MXene film by subjecting it to 10 MHz nanoscale nanoscale electromechanical vibration for a minute. Though the idea appears to be novel, it lacks sufficient evidence on the mechanistic aspects of the process. Following are some of the comments that may be useful in revising the manuscript.
1. What is the mechanism of removal of surface oxide layer on MXenes. Could 10 MHz vibration desorb oxy functional groups on Titanium sites? Or is it entire TiO2 getting desorbed off from the MXene surface. Due to low oxidation state of Titanium in Ti3C2Tz MXene, these phases are meta stable states and would get into stable states by atmospheric oxidation and passivation.
2. Surface oxide removal been demonstrated on a few micron thick MXene films. What about 10 nm MXene thin films? They are more prone oxidation compared to thick films. Also, surface plasmon peak of MXene transparent films can be monitored up on removal of surface oxide layer.
3. Has this technique applied to other MXene compostions. For example, Ti2CTx more prone to oxidation compared to, Ti3C2Tx MXenes. It would be great if authors test the proposed concept on 21 MXene compositions.
4. How the 4-probe electrical conductivity is affected for the SRBW recovered MXene film compared to pristine film.
5. The general accepted mechanism of pseudocapacitive behavior in MXenes is based on the protonation of oxy functional groups. How much control does SRBW technique provide in removing electrochemical inactive oxy functional groups without touching the active Ti-O surface functional groups.There is a shift in the redox peak maxima for the SRBW recovered MXene film compared to pristine MXene films. Derivative plots would be helpful in identifying the peak potentials. What kind of electrochemical performance in noted for the SRBW recovered MXene film in Li-ion containing organic electrolytes.
6. Nyquist impedance plots must be plotted in 1:1 scale and Warburg region is seen same for both pristine and SRBW recovered films unlike the voltammetry data at different scan rates. What is the reason in this case? Can the surface oxide removal process enhances the electrochemical surface area that is accessible to electrolyte ions?
7. By removal of surface oxide layers, the amount of titanium present in the given MXene samples would be reduced. How long this process could sustain. Do one need to repeat this process again and again for the surface oxide removal.

Reviewer #2 (Remarks to the Author):

Review of "Recovery of Oxidized Two-dimensional…." By Ahmed et al for Nature Comm

ASSESSMENT
The fundamental idea at the core of this paper (kicking off the oxides from MXene structures using vibration) is very clever and will certainly be of interest to the community. This is one of those ideas that I have batted around in group meeting before, so I am glad to see a research group finally try it and show that the approach is sound.

MAJOR ISSUES
- Can this method be applied to dispersed flakes rather than just films?
- It seems as though the experiment needs to be repeated several times: Oxidize, remove, oxidize, remove. How many times can this cycle occur before the film loses mechanical integrity?

MINOR ISSUES
- Abstract: "potential owing" – awkward grammar

- Abstract: “lifespan shortening” – awkward grammar
- Page 3: “Once formed, TiO_2, ….” – this entire sentence is awkward in English.
- Page 5: It is not standard to put “committee” talk into the results; this belongs in the methods.

Reviewer #3 (Remarks to the Author):

The authors have demonstrated a method (high frequency nanoscale electromechanical vibration) to remove the TiO_2 particles produced by oxidation of $Ti_3C_2T_x$ MXenes. Electrochemical performance is recovered after applying the SRBW method.

However, the method only removes the TiO_2 particles on the surface, but has little effect either on improving defect properties or on improving oxidation stability. In addition, the MXene sample used in the work is very limited. To prepare the oxidized sample, the authors just left the vacuum-filtrated MXene film in air ambient conditions for a month. When a MXene film is stored in a such an ambient condition, only a small fraction of layers at the surface are oxidized, and sublayers remain intact, i.e. the degree of oxidation is not large.

A similar effect (elimination of the oxidized parts and recovery of characteristics) can be obtained by simply applying heat treatment as the authors cited, thus it might not be very attractive for researchers to use the expensive and complicated process. The authors should present that SRBW method is much more powerful. For example, the authors should demonstrate the SRBW method is still effective for highly oxidized MXenes (oxidized in aqueous solution for a long time).

Specific Responses to the Comments of Reviewer 1

Authors have proposed a new strategy in recovering oxidized MXene film by subjecting it to 10 MHz nanoscale nanoscale electromechanical vibration for a minute. Though the idea appears to be novel, it lacks sufficient evidence on the mechanistic aspects of the process. Following are some of the comments that may be useful in revising the manuscript.

We sincerely thank the reviewer for their examination of our work and for their insightful remarks and recommendations. We have conducted further experiments and revised the manuscript accordingly to address their queries. We hope this is to the satisfaction of the reviewer and believe this to result in a stronger manuscript.

1. What is the mechanism of removal of surface oxide layer on MXenes. Could 10 MHz vibration desorb oxy functional groups on Titanium sites? Or is it entire TiO2 getting desorbed off from the MXene surface. Due to low oxidation state of Titanium in Ti3C2Tz MXene, these phases are meta stable states and would get into stable states by atmospheric oxidation and passivation.

In addition to the high-resolution x-ray photoelectron spectroscopic (XPS) analysis in the Ti 2p region that was reported in the original manuscript, we have now included more detailed XPS results including the oxygen functional groups to reveal the change in surface termination between the pristine, oxidised, and SRBW-treated samples. This is reported in Fig. S2 and Tables S5 and S6 in the Supporting Information where a comparison between the pristine and SRBW-treated samples indicate almost no variation in the surface termination, including the oxygen functional groups. This, together with the XPS analysis in the Ti 2p region and the x-ray diffraction (XRD) spectra, shows the amount of TiO_2 to be reduced following SRBW treatment, therefore confirming that the SRBW removes the oxide particles from the MXene surface.

We were however not able to quantify the surface terminations for the control oxidised sample as it is difficult to separate between the MXene's surface terminations and the –O and –OH groups adsorbed on TiO_2 since both have the same binding energy (Halim et al. *Appl Surf Sci* 362 (2016): 406; Halim et al. *RSC Adv* 8 (2018): 36785). As such, while there could be a possibility that the SRBW treatment also removes the oxygen functional groups from the titanium sites, we are unable to prove or disprove this conclusively at present to our best ability. We have added this discussion to the revised manuscript on pages 9 and 10.

2. Surface oxide removal been demonstrated on a few micron thick MXene films. What about 10 nm MXene thin films? They are more prone oxidation compared to thick films. Also, surface plasmon peak of MXene transparent films can be monitored up on removal of surface oxide layer.

The reviewer is correct that thin MXenes are more prone to oxidation. Following the reviewer's comment, we have now included results for thin (15 nm thick) MXene (Figs. S7 and S8 together with the XPS analysis in Tables S7 and S8), for which we confirm the possibility of successfully recovering the sample to levels similar to that for the thicker film (64% reduction in total oxide content), with almost no change in the surface terminations between the pristine (prior to oxidation) and SRBW-treated cases.

3. Has this technique applied to other MXene compostions. For example, Ti2CTx more prone to oxidation compared to, Ti3C2Tx MXenes. It would be great if authors test the proposed concept on 21 MXene compositions.

Given that there does not exist that specific number (21) of compositions of MXenes and since the reviewer provided the example of Ti_2CT_x, we understood from this context that the reviewer meant 2:1 MXene compositions. Based on their suggestion, we have therefore included an additional MXene that has a 2:1 composition, i.e., Mo_2CT_x. The results, with a recovery of 64% (Fig S8, Tables S7 and S8), are consistent with our observations for $Ti_3C_2T_z$.

4. How the 4-probe electrical conductivity is affected for the SRBW recovered MXene film compared to pristine film.

The conductivity of the SRBW-treated sample shows almost completely recovery (~97%) compared to the pristine sample. We have now added this to the revised manuscript on page 10.

5. The general accepted mechanism of pseudocapacitive behavior in MXenes is based on the protonation of oxy functional groups. How much control does SRBW technique provide in removing electrochemical inactive oxy functional groups without touching the active Ti-O surface functional groups. There is a shift in the redox peak maxima for the SRBW recovered MXene film compared to pristine MXene films. Derivative plots would be helpful in identifying the peak potentials. What kind of electrochemical performance in noted for the SRBW recovered MXene film in Li-ion containing organic electrolytes.

Since there is virtually no difference between the oxygen functional group surface terminations between the pristine and SRBW-treated samples (Fig. S2 and Tables S5 and S6), the shift in the peaks in the cyclic voltammograms (CV) between the samples is likely to be unrelated to the removal of the active Ti-O surface functional groups. Instead, we hypothesise that the presence of some TiO_2 residuals, following SRBW treatment, could be the main reason for the additional overall electrode resistance. Shifts in the redox peak maxima have similarly been attributed to the presence of TiO_2 in the literature—see, for example, Tang et al., *Angew Chem Int Ed* 58 (2019): 17849-17855.

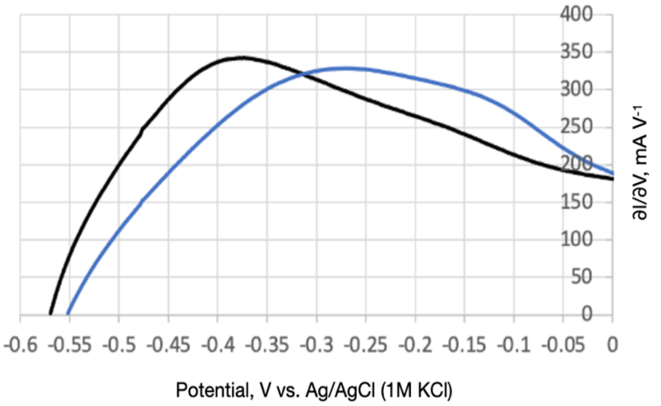

Derivative plot showing the anodic peak potentials for the pristine (blue curve) and SRBW-treated (black) $Ti_3C_2T_z$ MXene samples.

Following the reviewer's recommendation, an analysis of the derivative plots for the cyclic voltammetry at 10 mV s^{-1} shown above reveals the charging CV profiles for the anodic reaction peaks to occur at -

0.28 V and -0.37 V for the pristine and the SAW-treated samples, respectively. The approximate 0.1 V shift in the redox reaction towards the negative potential is associated with the electrode overpotential that is likely due to the increase in overall resistance as a consequence of some residual TiO_2 particles on the SRBW-treated MXene film (the initial TiO_2 content in the pristine and oxidised samples are 17% and 79%, respectively, compared to that for the SRBW-treated sample, which is 31%). In addition to the aforementioned reference, this postulation is supported by the observation that the shift in the redox peaks were also observed in the fully oxidised electrode (Figs. 4B and S5B).

Since there have been many studies on the capacitance of MXene in different electrolytes, including lithium ions (see, for example, Dall'Agnese et al. *J Power Sources* 306 (2016): 510-515), we have limited our focus to the novel aspects of the present work, which is to show the potential of the SRBW treatment to recover oxidised freestanding MXene films. The electrochemical performance we show therefore were only meant to show recovery of the film.

6. Nyquist impedance plots must be plotted in 1:1 scale and Warburg region is seen same for both pristine and SRBW recovered films unlike the voltammetry data at different scan rates. What is the reason in this case? Can the surface oxide removal process enhances the electrochemical surface area that is accessible to electrolyte ions?

We agree that the Nyquist plot should be plotted in 1:1 scale and have updated Fig. 4E accordingly. As the SRBW treatment does not fully remove all of the surface oxides, leading to slightly higher overpotential in the CV (see point 5 above as well as Tang et al., *Angew Chem Int Ed* 58 (2019): 17849-17855), it is also likely, as the reviewer hinted, that the small amount of TiO_2 particles left on the MXene surface following treatment could indeed lead to an enhancement in the electrochemical surface area accessible to the electrolyte ions. This is because the TiO_2 particles can act as a spacer between the MXene sheets to avoid compact restacking of the sheets, which is also consistent with our XRD results. Consequently, the electrode has then more accessible channels for electrolyte ions to penetrate and adsorb onto (see, for example, Pazniak, et al., *ACS Appl Nano Mater* 3 (2020): 3195-3204 and Zhang et al., *Inorg Chem Front* 7 (2020): 1205-1211).

7. By removal of surface oxide layers, the amount of titanium present in the given MXene samples would be reduced. How long this process could sustain. Do one need to repeat this process again and again for the surface oxide removal.

Following the reviewer's comment, we have conducted additional experiments and now demonstrate that it is possible to sustain multiple (at least 3) oxidation/SRBW treatments while maintaining the film's integrity, as shown in Fig. S6.

Specific Responses to the Comments of Reviewer 2

The fundamental idea at the core of this paper (kicking off the oxides from MXene structures using vibration) is very clever and will certainly be of interest to the community. This is one of those ideas that I have batted around in group meeting before, so I am glad to see a research group finally try it and show that the approach is sound.

We sincerely thank the reviewer for their careful scrutiny of our work, and for their very positive comments. We have carefully taken into account their queries and recommendations, and have revised the manuscript accordingly. We hope that this is to the reviewer's satisfaction and that it has led to a stronger paper.

MAJOR ISSUES

- Can this method be applied to dispersed flakes rather than just films?

Following the reviewer's question, we have now conducted a new set of experiments to show that the method equally applies when flakes are oxidized in solution (Fig. S6).

- It seems as though the experiment needs to be repeated several times: Oxidize, remove, oxidize, remove. How many times can this cycle occur before the film loses mechanical integrity?

We thank the reviewer for the suggestion and have conducted additional experiments to demonstrate that it is possible to sustain multiple (at least 3) oxidation/SRBW treatments (Fig. S6) without any visible change in the mechanical integrity.

MINOR ISSUES

- Abstract: "potential owing" – awkward grammar
- Abstract: "lifespan shortening" – awkward grammar
- Page 3: "Once formed, TiO2, …." – this entire sentence is awkward in English.
- Page 5: It is not standard to put "committee" talk into the results; this belongs in the methods.

We thank the reviewer for their careful reading of our manuscript and have addressed the first three items above in the revised manuscript. Regarding the last item, we should point out that the reference to the Joint Committee on Powder Diffraction Standards (JCPDS) pertains to the reference XRD data for anatase and rutile TiO_2 that we compare our results against. As such, it is not a method but part of the discussion and analysis of the results. We have now added a citation to the JCPDS in the revised manuscript.

Specific Responses to the Comments of Reviewer 3

The authors have demonstrated a method (high frequency nanoscale electromechanical vibration) to remove the TiO_2 particles produced by oxidation of $Ti_3C_2T_x$ MXenes. Electrochemical performance is recovered after applying the SRBW method.

We sincerely thank the reviewer for their examination of our work. We have conducted further experiments and revised the manuscript accordingly to address their queries. We hope this is to the satisfaction of the reviewer and believe this to result in a stronger manuscript.

However, the method only removes the TiO_2 particles on the surface, but has little effect either on improving defect properties or on improving oxidation stability. In addition, the MXene sample used in the work is very limited. To prepare the oxidized sample, the authors just left the vacuum-filtrated MXene film in air ambient conditions for a month. When a MXene film is stored in a such an ambient condition, only a small fraction of layers at the surface are oxidized, and sublayers remain intact, i.e. the degree of oxidation is not large.

We agree with the reviewer that the method does not improve defect properties or enhance oxidation stability. We however did not claim that that the SRBW treatment can do this, which other works have already been shown to be capable of. Instead, we have focussed on the ability of the acoustic vibration to facilitate recovery of oxidised MXene films, and, additionally in the revised manuscript, dispersed MXene flakes oxidized in solution, which we believe to be novel and hence have focussed the work on this aspect.

Following the reviewer's comment above, we also show the method additionally works for MXene films rapidly oxidised under heat. In addition, we also demonstrate that the process is viable for multiple treatments (at least 3, as shown in Fig. S6), without any visible change in the film's mechanical integrity.

A similar effect (elimination of the oxidized parts and recovery of characteristics) can be obtained by simply applying heat treatment as the authors cited, thus it might not be very attractive for researchers to use the expensive and complicated process. The authors should present that SRBW method is much more powerful. For example, the authors should demonstrate the SRBW method is still effective for highly oxidized MXenes (oxidized in aqueous solution for a long time).

We have now included additional data to demonstrate the broader appeal of the technique, for example, for molybdenum-based MXene flakes (Fig. S8 and Tables S7 and S8) that are considerably thinner (~15 nm, Fig. S7), as well as flakes that have been oxidized while dispersed solution in addition to being able to conduct multiple SRBW-recovery cycles without detriment to the integrity of the material (Fig. S6).

We should point out that our method is not as expensive and complicated as the reviewer might have perceived. In fact, it is arguably as simple as, if not simpler than, the heat treatment that the reviewer alluded to. In place of heat, we simply vibrate the film. Moreover, as pointed out in the last paragraph of the Conclusions in the original manuscript, our platform is considerably less costly than the heat treatment given the low cost of the devices (around US$1 each by exploiting the economies-of-scale associated with mass nanofabrication) and their low operation costs (low power requirement of around 1 W). This, together with the rapid procedure (approximately 1 min) allows for the possibility of scaling the technique through massive parallelisation for high throughput operation. This is in contrast to the large and expensive infrastructure necessary to produce the requisite high vacuum and temperatures for the heat treatment, which is also a lengthy process (0.5-45 hrs of annealing time).

Reviewer comments, second round review:

REVIEWER COMMENTS

Reviewer #1 (Remarks to the Author):

My comments have been addressed satisfactorily. I recommend for publication

Reviewer #2 (Remarks to the Author):

The authors have addressed my major concerns.

MINOR EDITS:

Look at the following sentence.

"Multiple SRBW restoration treatments following re-oxidation of the film are also possible without detriment to the film's integrity, as seen by the results in Fig. S6 in the Supporting Information following three successive oxidation and SRBW-treatment cycles, wherein we also demonstrate that the treatment also works irregardless of the way by which the sample is oxidized (dispersed in solution, as opposed to being oxidized as a film; in addition, the second and third oxidation steps were also carried out differently by heating the film at 100 °C for 12 h)."

This is a long, run-on sentence with multiple grammar problems. Also, "irregardless" is not a word.

Reviewer #3 (Remarks to the Author):

The authors concluded that the SRBW removes the TiO_2 particles from the surface of MXene. However, to meet high standards for publication in Nature comm., a higher level of technology is required rather than removing TiO_2 particles on the surface using physical force, for example, reducing the oxidized MXene to unoxidized state (TiO_2 to Ti_3C_2).

Leaving the MXene film at 100°C for 12 hours only oxidizes the outermost surface of the MXene, not deep inside, but rather contributes to the removal of the H_2O molecules present between layers. MXene film does not oxidize well unless it is in high moisture conditions or dispersion in DI-water. Whether it is repeated twice or three times, the degree of phase transformation of Ti_3C_2 MXene is not much.

XPS results of the samples oxidized three times are shown in Figure S6, and the degree of oxidation is not large. There is still a large Ti-C peak around 455 eV corresponding to Ti-C. Even in MXene that is little oxidized (even pristine MXene), after a few days, the outermost surface of the MXene has a low level of TIO_2 nanoparticles.

In addition, if SRBW technology is only removing oxide particles on the surface of MXene, there are several alternative techniques, thus it seems not to have a high impact. For example, since the outermost surface of MXene is very easily oxidized, before taking XPS, it is sometimes required to use a sputtering to remove impurities including TiO2. (Applied Surface Science 362 (2016) 406–417) In addition, TiO2 particles formed on the surface can be removed through heat treatment (J. Mater. Chem. A, 2020, 8, 573).

Therefore, this study needs to be redesigned to have a huge impact. Improving the oxidation stability of MXenes in the aqueous phase is more urgent and more difficult than in the film.

It is much more meaningful and required to proceed with the highly oxidized MXene sample, which has been oxidized in an aqueous solution. For example, put the MXene dispersion in DI-water in an oven at 70-80 °C for several weeks to get highly oxidized MXenes. If the same effect can be seen with SRBW, it will have a much greater impact than the present work.

Specific Responses to the Comments of Reviewer 2

"Multiple SRBW restoration treatments following re-oxidation of the film are also possible without detriment to the film's integrity, as seen by the results in Fig. S6 in the Supporting Information following three successive oxidation and SRBW-treatment cycles, wherein we also demonstrate that the treatment also works irregardless of the way by which the sample is oxidized (dispersed in solution, as opposed to being oxidized as a film; in addition, the second and third oxidation steps were also carried out differently by heating the film at 100 °C for 12 h)."

This is a long, run-on sentence with multiple grammar problems. Also, "irregardless" is not a word.

We thank the reviewer for their careful reading of our revised manuscript and for pointing this out. We have now shortened the sentence and corrected the typo.

Specific Responses to the Comments of Reviewer 3

We thank the reviewer for re-evaluating our work. We have carefully studied their comments and would like to offer a clarification to each point that they have raised below.

The authors concluded that the SRBW removes the TiO2 particles from the surface of MXene. However, to meet high standards for publication in Nature comm., a higher level of technology is required rather than removing TiO2 particles on the surface using physical force, for example, reducing the oxidized MXene to unoxidized state (TiO2 to Ti3C2).

When MXene is oxidized, Ti_3C_2 is converted to TiO_2 and C. The TiO_2 then remains and C dissolves to form CO and CO_2 (Huang et al., *Inorg Chem* 58, 1958, 2019):

$$2Ti_3C_2O_2 + 11H_2O \leftrightarrow 6TiO_2 + CO + CO_2 + 2CH_4 + 7H_2$$

$$2Ti_3C_2(OH)_2 + 11H_2O \leftrightarrow 6TiO_2 + CO + CO_2 + 2CH_4 + 9H_2$$

As such, we do not see how it is possible to reduce TiO_2, being very stable, to Ti_3C_2 as the reviewer suggests.

Leaving the MXene film at 100°C for 12 hours only oxidizes the outermost surface of the MXene, not deep inside, but rather contributes to the removal of the H2O molecules present between layers. MXene film does not oxidize well unless it is in high moisture conditions or dispersion in DI-water. Whether it is repeated twice or three times, the degree of phase transformation of Ti3C2 MXene is not much.

XPS results of the samples oxidized three times are shown in Figure S6, and the degree of oxidation is not large. There is still a large Ti-C peak around 455 eV corresponding to Ti-C. Even in MXene that is little oxidized (even pristine MXene), after a few days, the outermost surface of the MXene has a low level of TIO2 nanoparticles.

Firstly, there is a misunderstanding here. We actually carried out partial oxidation at 100°C for 12 hours on dispersed MXenes in aqueous solution (and not on dry films as the reviewer had indicated in their comment above). This was described in the oxidation procedure in the methods section on page 18. We have now revised the wording on page 12 to make this clearer.

Secondly, we respectfully disagree with the reviewer that the degree of oxidation is not large. Figure S6, which the reviewer refers to above, pertains to multiple oxidation and recovery cycles, which was requested by another reviewer. This was however not the main finding of our work and was simply added on request of that reviewer to demonstrate the possibility of multiple recovery cycles. The main finding can be seen from Fig. 3 in the main manuscript and Table S2 wherein we show, for example, that the degree of oxidation increases from 17% for the pristine sample to 79% for the oxidized sample. While not completely oxidizing the sample (complete oxidation would not have been useful to show anyway since there is no way to restore completely (or near complete) oxidized samples as there would not be any more MXene left (reduction of TiO_2 to Ti_3C_2 is not possible)), this is still fairly substantial to demonstrate the efficacy of the acoustic mechanism in removing the oxide layer from the

surface of the MXene where the oxidation level can be seen to decrease to 30% for the recovered sample).

In addition, if SRBW technology is only removing oxide particles on the surface of MXene, there are several alternative techniques, thus it seems not to have a high impact. For example, since the outermost surface of MXene is very easily oxidized, before taking XPS, it is sometimes required to use a sputtering to remove impurities including TiO2. (Applied Surface Science 362 (2016) 406–417) In addition, TiO2 particles formed on the surface can be removed through heat treatment (J. Mater. Chem. A, 2020, 8, 573).

We respectfully disagree with the reviewer on this point. As clarified above, the recovery of completely oxidized monolayer MXenes is not possible. Our proposed method for recovering partially oxidized MXenes by removal of the TiO_2 surface layer solves a well-known and widespread problem hampering the industry where films left out under ambient environments form surface oxide layers over time, significantly degrading their performance (we note too that we had shown in the previous revised manuscript that our method is also suitable for thin MXene films down to 15 nm in thickness, therefore broadening the practical utility of the technique).

The other methods for TiO_2 removal that the reviewer alludes to are either significantly more complex, cumbersome (e.g., necessity for vacuum or Ar) and costly (sputtering), or, have the propensity to degrade (heating to 450 ^oC), re-oxidise (due to leaks in the inert atmosphere) or alter the surface termination (annealing) of the material, compared to the facile use of vibration in the present technique, which is not only simple but also extremely quick (1 min) and low cost (approx. $1/device). Moreover, we note that with sputtering to remove the oxide layer in the work that was cited (of which two of us were authors), only a very slight reduction in the oxide level of around 6% was observed, as opposed to up to around 60% reduction with our method.

Therefore, this study needs to be redesigned to have a huge impact. Improving the oxidation stability of MXenes in the aqueous phase is more urgent and more difficult than in the film.

It is much more meaningful and required to proceed with the highly oxidized MXene sample, which has been oxidized in an aqueous solution. For example, put the MXene dispersion in DI-water in an oven at 70-80 °C for several weeks to get highly oxidized MXenes. If the same effect can be seen with SRBW, it will have a much greater impact than the present work.

As mentioned above, we had already conducted experiments on MXene dispersions in DI-water in the previously revised manuscript. We however did not oxidize this for several weeks as the reviewer suggests, since that would have led to total (or near total) oxidation of the dispersed MXene flakes completely into TiO_2 particles, which cannot be reduced back to Ti_3C_2 for the aforementioned reasons.

To belabor the point, we note that unlike a film, monolayer (there is no practical reason to disperse multilayers in solution) MXene flakes dispersed in aqueous solution are oxidized

individually. As such, TiO_2 crystals will also grow within the flake if it is highly oxidized beyond just a surface oxide layer. Removing these, even if it were possible (see our clarification above that it is not possible to reduce TiO_2 to Ti_3C_2), will create pores in the flake, destroying the integrity of the material.
